# Genomic divergence landscape in recurrently hybridizing *Chironomus* sister taxa suggests stable steady state between mutual gene flow and isolation

**DOI:** 10.1002/evl3.204

**Published:** 2020-11-06

**Authors:** Dennis Schreiber, Markus Pfenninger

**Affiliations:** ^1^ Department of Molecular Ecology Senckenberg Biodiversity and Climate Research Centre Frankfurt am Main 60325 Germany; ^2^ Institute for Molecular and Organismic Evolution Johannes Gutenberg University Mainz 55128 Germany; ^3^ LOEWE Centre for Translational Biodiversity Genomics (LOEWE‐TBG) Frankfurt am Main 60325 Germany

**Keywords:** Admixture inference, islands of divergence, reproductive isolation, speciation

## Abstract

Divergence is mostly viewed as a progressive process often initiated by selection targeting individual loci, ultimately resulting in ever increasing genomic isolation due to linkage. However, recent studies show that this process may stall at intermediate stable equilibrium states without achieving complete genomic isolation. We tested the extent of genomic isolation between two recurrently hybridizing nonbiting midge sister taxa, *Chironomus riparius* and *Chironomus piger*, by analyzing the divergence landscape. Using a principal component‐based method, we estimated that only about 28.44% of the genomes were mutually isolated, whereas the rest was still exchanged. The divergence landscape was fragmented into isolated regions of on average 30 kb, distributed throughout the genome. Selection and divergence time strongly influenced lengths of isolated regions, whereas local recombination rate only had minor impact. Comparison of divergence time distributions obtained from several coalescence‐simulated divergence scenarios with the observed divergence time estimates in an approximate Bayesian computation framework favored a short and concluded divergence event in the past. Most divergence happened during a short time span about 4.5 million generations ago, followed by a stable equilibrium between mutual gene flow through ongoing hybridization for the larger part of the genome and isolation in some regions due to rapid purifying selection of introgression, supported by high effective population sizes and recombination rates.

**Impact Summary**

The process of speciation has fascinated biologists from early on. Prevailing theory suggested that gene flow among populations is the main obstacle for their divergence. Recently, it became clear that speciation with gene flow is possible under certain circumstances. However, it remains unclear how the divergence process proceeds in time, how widespread the phenomenon is, and whether it always and inevitably leads to complete isolation. Comparing the genomes of individuals of two regularly hybridizing sister taxa of nonbiting midges, we could show that they diverged during a short period millions of generations ago. Their divergence process apparently ceased before the entire genome was mutually isolated. The taxa remain distinct since, even though they share most of their genome. Our findings thus extend our view of the nature of species and the temporal dynamics of their divergence and describe novel approaches to analyze both current and past divergence processes.

If selection acts simultaneously in disparate directions, favoring different phenotypes, divergence processes are initiated (Rundle and Nosil 2005). However, such divergence processes may not need to affect the genome as a whole but rather lead to heterogeneous genomic differentiation (Martin et al. 2013; Feulner et al. 2015; Duranton et al. 2018; Ravinet et al. 2018). When selection targets individual loci underlying the favorable phenotype, only these as well as linked loci will diverge (Via and West 2008). These diverging genomic regions under selection are shielded from gene flow by locally reduced recombination rates (Via 2012), whereas the predominant part of the genome remains freely exchangeable among emerging taxa (Wu 2001; Feder et al. 2012b). This process is called divergence hitchhiking (DH; Via and West 2008) and results in heterogeneous genomic differentiation patterns with small genomic regions of elevated differentiation referred to as “islands of divergence” (Turner et al. 2005). Such patterns of genomic divergence are already well documented across a wide range of taxa: Studies on *Anopheles gambiae* (Turner et al. 2005) as well as *Ficedula* species (Ellegren et al. 2012) revealed “islands of divergence” often located near telomeres and centromeres, regions with reduced recombination rates (Chen et al. 2008). In *Heliconius* species, divergence peaks were found in presumed regulatory regions associated with wing patterns that are commonly involved in mate recognition and therefore under sexual selection (Nadeau et al. 2012). At the same time, *Heliconius* species experience frequency‐dependent selection on local patterns due to their role in predator avoidance (Nadeau et al. 2012). Taking the temporal course of divergence into account, a study on *Dicentrarchus labrax* found several regions responsible for initial reproductive isolation (RI) evolved during allopatry in the two lineages that were eroded by gene flow during secondary contact (Duranton et al. 2018).

Genomic divergence is thus a progressive process, initiated by selection targeting local genomic regions and expanding from there over time, eventually leading to speciation with gene flow (Feder et al. 2012b; Via 2012). Feder et al. (2012b) recognized four phases in this process. (i) During the first phase, direct selection targets (independent) loci inducing divergence, whereas the rest of the genome is still exchanged by gene flow. (ii) The second phase is characterized by DH elevating divergence in the proximity of loci under selection. (iii) In the third phase, DH is either already widespread throughout the genome or the selection is strong enough to decrease gene flow on a genome‐wide scale. This leads to the fixation of further divergent loci resulting in “continents of divergence.” (iv) This so‐called genome hitchhiking (GH) eventually leads to a fourth phase in which gene flow increasingly ceases throughout the genome.

However, this sequence of events does not appear to be an inevitably progressive process. Several empirical studies (Schwenk et al. 2000; Faure et al. 2009; Canestrelli et al. 2017) as well as theoretical models (Yeaman and Whitlock 2011; Flaxman et al. 2014; Rafajlović et al. 2016) suggest the possibility of intermediate constant equilibrium states where certain parts of the genome remain diverged (“islands” or “continents of divergence”), whereas others are freely exchanged among closely related species without ever reaching complete genomic isolation (GI). Theoretical prerequisites for such a migration‐selection‐drift equilibrium, effectively freezing the divergence process in some phase of the Feder et al. model (2012b), are beneficial mutations locally connected through linkage and either reduced recombination or increased selection in genomic regions differing in divergent taxa (Yeaman and Whitlock 2011; Rafajlović et al. 2016). However, it remains unclear how frequent such equilibria among hybridizing taxa occur.

We investigated the state of divergence and the possibility of a stable migration‐selection‐drift equilibrium in two cryptic, nonbiting midges sister taxa *Chironomus riparius* (Meigen, 1803) and *Chironomus piger* (strenzke, 1956). Both of these multivoltine taxa are found in freshwater habitats such as small streams, ditches, and puddles (Pfenninger and Nowak 2008) throughout the temperate Holarctic (Strenzke 1957; Oppold 2017) and spend most of their life in one of four aquatic larval stages before they pupate, emerge, and mate in swarms (Armitage et al. 1995). The two taxa often co‐occur throughout their largely overlapping distribution ranges with no known differential centers of distribution (Kiknadze et al. 1991; Gunderina and Salina 2003; Petrova et al. 2014; Pedrosa et al. 2017). Usually, one taxon prevails due to differential adaptation to ecological niches (Pfenninger and Nowak 2008). *Chironomus piger* is known, for example, to tolerate higher nitrite pollution and salt concentrations (Pfenninger and Nowak 2008), which have negative impact on larval development in both taxa (Haas and Strenzke 1957; Neumann et al. 2001). Thus, *C. piger* usually predominates in lentic waterbodies near agricultural landscapes (Foucault et al. 2019), whereas *C. riparius* prevails in slow‐flowing streams.

*Chironomus riparius* and *C. piger* differ remarkably in genome size: the *C. riparius* genome is around 30% larger compared to the sister taxon. This increased genome size of *C. riparius* can be explained in large part by the expansion of a certain transposable element, the so‐called *Cla‐element*. In addition, this element has been suggested to be involved in the initiation of the speciation process (Schmidt 1984), which was estimated to have occurred about 1.3–1.8 million years ago (Schmidt et al. 2013). Karyotype comparisons with more distantly related *Chironomus* species further support the assumption that the smaller genome of *C. piger* is the ancestral state (Keyl 1965). The suspicion that *Cla‐elements* are drivers of divergence is strengthened by strong selection against heterozygous population‐specific insertions in interpopulation crosses of *C. riparius* (Oppold et al. 2017). Despite this obvious genomic difference, hybridization between the two taxa has been observed both in the lab (Foucault et al. 2019) and in nature (Petrova et al. 2014; Pedrosa et al. 2017; Foucault et al. 2019). However, substantial pre‐ and postzygotic RI barriers, namely, optical swarming markers (Miehlbradt and Neumann 1976) and hybrid dysgenesis syndromes (Hägele 1984, 1999; Armitage et al. 1995), lead to generally lower fitness in all (back)crossing directions (Foucault et al. 2019). This results in strong selection against introgression reflected in the low abundance of recently admixed individuals in the field (Pedrosa et al. 2017; Foucault et al. 2019). Given the estimated divergence time of about 1.5 million years ago (Schmidt et al. 2013), which corresponds to at least 9 million generations (Oppold et al. 2016), it is remarkable that hybridization is still ongoing. Strong divergent selection, long‐standing divergence, and persistent hybridization make the sister taxa *C. ripari*us and *C. piger* a promising system to investigate the extent of GI as well as the state of the divergence process, in particular the possibility of a stable selection‐migration‐drift equilibrium.

## Materials and Methods

### SAMPLING, SEQUENCING, AND DATA PROCESSING

We whole genome sequenced 36 individual *Chironomus* specimen from five different sites across Europe (Fig. S1; Table S1). Sequencing was performed on an Illumina HiSeq4000 platform, using the KAPA library preparation kit, resulting in around 25× coverage 150 bp paired‐end reads per sample.

After quality checking, trimming, and mapping to the latest *C. riparius* reference genome (Schmidt et al. 2020), single nucleotide polymorphisms (SNPs) were called with GATK (DePristo et al. 2011). The filtered VCF file contained 5,496,457 variable positions. For further details on tools, options, parameters, and commands, see Supporting Information 1.1.

### ADMIXTURE ANALYSES

Using bcftools version 1.9 (Li 2011), we filtered out strongly linked loci with *r* ^2^ > 0.8 in 100‐kb windows to prune the dataset for SNPs in linkage disequilibrium (LD; Alexander et al. 2009; Baran et al. 2013). This retained 900,345 loci. ADMIXTURE version 1.3.0 (Alexander et al. 2009) was used to infer ancestry proportions based on maximum likelihood estimations using *K* = 2 with default settings. In addition, we used the R (R Core Team 2017) package factoextra (Kassambara and Mundt 2017) to calculate a principal component analysis (PCA) on unlinked SNPs successfully called in all individuals.

### IDENTIFICATION OF MUTUALLY ISOLATED GENOMIC REGIONS

We developed a PCA‐based approach for identifying genomic regions that are mutually isolated between two hybridizing taxa, which requires no a priori known admixture‐free reference individuals for the respective species. PCA is not based on any particular population genetic model (Zheng and Weir 2016) and is therefore widely used to infer major trends in individual‐based SNP data.

PCA was performed on all SNPs successfully genotyped in all individuals using the R (R Core Team 2017) package factoextra (Kassambara and Mundt 2017). The first PC describes the largest proportion of variance in the data, meaning it splits the most divergent units, that is, the described taxa as identified by DNA barcoding (Table S1). On this axis, we identified SNPs whose cumulative sum of factor scores increased linearly (species separating SNPs [sepSNPs]; Fig. S2). The remaining SNPs contributed only negligibly to the variance among species (Fig. S2) and were flagged as residual SNPs.

Isolated genomic areas were defined as nonoverlapping windows containing more than statistically expected sepSNPs (*χ*²‐test with Benjamini‐Hochberg FDR correction for multiple testing). To retain sufficient statistical power, we chose a window size containing at least 60 SNPs in more than 95% of the windows (i.e., 10 kb). Windows smaller than the chosen size arose from windows at scaffold ends.

Additionally, we estimated the fixation index (*F*_ST_; Weir and Cockerham 1984) in the same 10‐kb windows using VCFtools version 0.1.15 (Danecek et al. 2011). We calculated the point biserial correlation between *F*_ST_ values per window and the classification as isolated or nonisolated based on our PCA approach.

### INFLUENCE OF RECOMBINATION RATE ON ISOLATION

We estimated mean LD for each 10‐kb window using the maximum likelihood method on Pool‐Seq data of Feder et al. (2012a) on a published pool from one of the populations sampled here (ENA project ERP115516, sample ERS4040036). This method exploits the haplotype information contained in (paired) reads and yields accurate estimates if LD decay to background levels is shorter than the read length (Feder et al. 2012a), which was 250 bp here (see Fig. S3). The extension to the entire species assumed that the recombination landscape is highly correlated within species (Samuk et al. 2020).

### GENE ONTOLOGY TERM ENRICHMENT ANALYSES

We conducted gene ontology (GO) term enrichment analyses on the categories Biological Process (BP) and Cellular Component (CC) on genes in isolated and nonisolated regions as well as on positively selected genes (see below). The analyses on 7951 GO‐annotated genes were carried out using the R package topgo (Alexa and Rahnenführer 2018). Only terms with more than five annotated genes were considered. See Supporting Information 1.4 for details.

### HISTORIC DIVERGENCE DYNAMICS

According to the DH concept, the size of isolated genomic regions should increase with time until finally the entire genome is isolated (Feder et al. 2012b). This yields three testable predictions:

i) As divergence islands tend to increase in size over time, their mean length should be larger than islands resulting from randomly distributing the same number of identified isolated windows over the genome (see Supporting Information 1.5).
ii) In most models of speciation with gene flow, GI inevitably increases over time until it is complete or reaches an equilibrium state. Isolated regions should therefore increase both in number and size over time and divergence time estimates of isolated region should extend to the present (or when full GI was reached) or until a stable steady‐state equilibrium is reached. To infer the isolation dynamics, we used the temporal information contained in the divergence time estimates for each isolated 10‐kb window (Supporting Information 1.5). We then simulated five plausible divergence scenarios with SIMCOAL (Excoffier 2000; see supplementary code in Supporting Information 1) and inferred the best‐fit scenario to the empirical divergence time distribution of isolated windows using an approximate Bayesian computation (ABC) inference framework (see Supporting Information 1.5 for details):
  a) Continuous divergence: Divergence is continuously acting over the entire time span with the same number of windows diverging per temporal bin.
  b) Early eruption, fading out: After the initial, early divergence of a few windows, the number of diverging windows increased very fast to eventually slow down but continue until today.
  c) Escalating divergence: Divergence started early with few windows and continuously increases over time, both in number and size of divergent windows.
  d) Episodic divergence: Divergence started with a few windows, increased fast, peaks at the mean observed divergence time, and completely ceased again.
  e) Instantaneous divergence: All divergence happened at once, at the mean observed divergence time.

**Figure 1:**
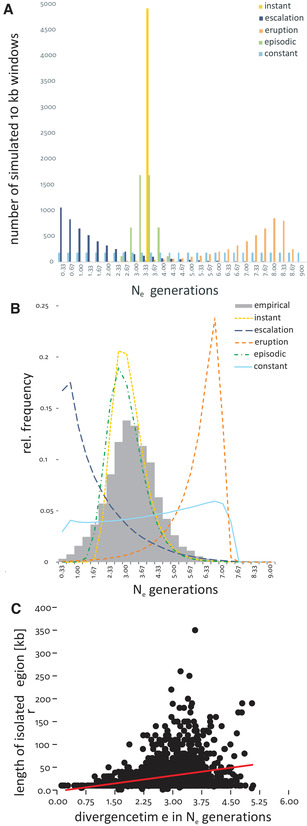
(A) Graphical representation of the divergence models tested. The bars show the number of isolated windows simulated for each time slice (in *N*_e_ generations; *N*_e_ = 1.5 × 10^6^) of the respective model in each of 100,000 replicates. The models are differentiated by color:instant, yellow; escalation, dark blue; eruption, orange; episodic, green; constant, light blue. (B) Comparison of observed frequency distribution of estimated divergence times (gray bars) with mean estimated divergence times of a subset of 100 replicates for each model (colored lines, color coding as before). (C) Relation between estimated mean divergence time and length of isolated region. Albeit significant, the positive relation (red line) explained very little of the variation (*η*² = 0.063).

The respective simulated frequency distributions for each model are depicted in Figure 1A. Please note that the simulations focused on currently diverged regions. All scenarios therefore include the possibility of secondary contact after an allopatric phase of divergence.

iii) If divergence islands grow with time, their size should be roughly proportional to their estimated divergence time. Additionally, the variance in size not explained by divergence time is expected to result from a reduction of gene flow by selection and physical linkage between pairs of SNPs (Wu 2001). To test this hypothesis, we joined adjacent isolated windows to isolated regions, calculated their length, and correlated it to the respective mean divergence time, strength of selection, and physical linkage (see Supporting Information 1.5 for details).

### PROCESSES DRIVING DIVERGENCE

We calculated Tajima's *D* (TD) for each isolation window of each taxon separately with MEGA X (Kumar et al. 2018) and compared the resulting distribution with a random sample of 1000 nonisolated windows in each taxon. Isolated windows with TD values falling beyond the upper or lower 95% confidence limits of the nonisolated windows distribution were considered as driven by nonneutral processes (Feulner et al. 2015). We considered outlier windows with significantly negative TD in both taxa as under divergent selection, while occurring in only a single taxon as positively selected in the respective taxon (Pfenninger et al. 2015).

We also evaluated the influence of the *Cla* elements on divergence. It is known that these repeat elements are enriched in heterochromatic regions (Schmidt et al 2020). As these are the regions that could not be resolved in the current genome assembly, scaffold ends tend to neighbor the clusters of *Cla* elements. To test for potential influence of *Cla* elements on divergence, we analyzed whether isolated regions were more often closer to scaffold ends than expected by chance.

To relate adaptive protein evolution to the divergence process, we extracted the coding sequence of all genes in the isolated regions. Using PopGenome (Pfeifer et al. 2014), we calculated the approximate version of the McDonald‐Kreitman test (MKT). Significance was assessed with a Fisher's exact test. For all significant genes, we calculated the neutrality index and the proportion of divergent SNPs fixed by positive selection (α; Smith and Eyre‐Walker 2002). For comparative reasons only, we did the same for genes in nonisolated regions, even though the MKT test assumption of divergence was by definition not met (Supporting Information 1.6).

## Results

### GENOME‐WIDE (RECENT) ANCESTRY INFERENCE

Both ADMIXTURE (Alexander et al. 2009) and a PCA approach confirmed the DNA barcoding‐based taxa assignment to *C. riparius* and *C. piger* and did not find indications of recent admixture (Table S1; Fig. S5). ADMIXTURE estimated admixture proportions below 1% across all samples. The PCA clustered the individual samples according to the a priori assignment and did not find any intermediate individuals (Fig. S6).

### CURRENT STATE OF MUTUAL GI

**Figure 2:**
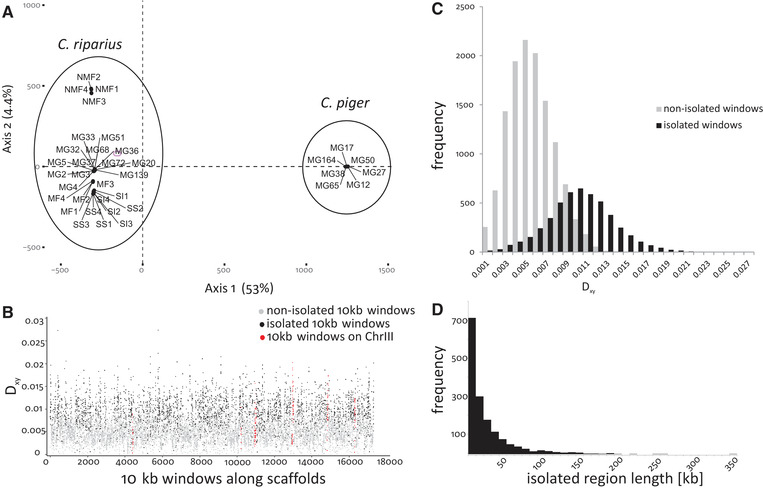
Identification of isolated windows and genomic isolation landscape. (A) Principal component analysis on all SNPs. Axis one, representing 53% of total variation, separated the two species as identified from microsatellite and mitochondrial marker loci. SNPs with high correlation to this axis were identified as species separating SNPs (sepSNPs). (B) Distribution of divergence estimates (*D_xy_*) over the genome (10‐kb windows with a significant overrepresentation of sepSNPs are marked in black, all others in gray). Isolated as well as nonisolated windows on scaffolds known to be on chromosome III are marked in red. (C) Frequency distribution of *D_xy_* estimates for isolated (black bars) and nonisolated windows (gray). (D) Frequency distribution of the length of isolated regions (i.e., joining adjacent isolated 10‐kb windows).

The PCA for identification of highly differentiated genomic regions was calculated based on the entire genotype dataset containing 3,270,391 SNPs successfully genotyped for all samples (Fig. 2A). PC1 explained 53% of variance and split *C. riparius* from *C. piger*. PC2, explaining 4.4% of variance, reflected intraspecific divergence of *C. riparius* (Fig. 2A). Cumulative locus factor scores of the 700,000 most contributing positions explained 90.88% of the variance of PC1 (sepSNPs). Using FDR‐corrected *χ*²‐tests to identify 10‐kb windows with significantly accumulated sepSNPs, 4917 out of 18,203 windows were identified as isolated (Supporting Information 2). These isolated windows were spread over the entire genome (Fig. 2B) and characterized by increased sequence divergence (*D_xy_*) compared to nonisolated ones (Figs. 2B and 2C). Joining immediately adjacent isolated windows reduced this number to 1626 isolated regions ranging from 1166 bp to 350 kb length (Fig. 2D). Isolated regions were found on 488 of 752 scaffolds of the reference genome.

Our PCA approach estimated thus 28.44% of the genome to be mutually isolated. F*_ST_* values were positively correlated with isolated regions identified by the PCA approach (Point‐biserial correlation, *r*_pb_ = 0.74, *P* < 0.001).

Isolated regions were also significantly further away from scaffold ends and thus from large *Cla* clusters than is expected by chance (empirical distance: 140 ± 138 kb [mean ± SD], simulated distance: 59 ± 1.3 kb [mean ± SD], *P* < 0.001).

### CHARACTERIZATION OF ISOLATED WINDOWS

**Figure 3:**
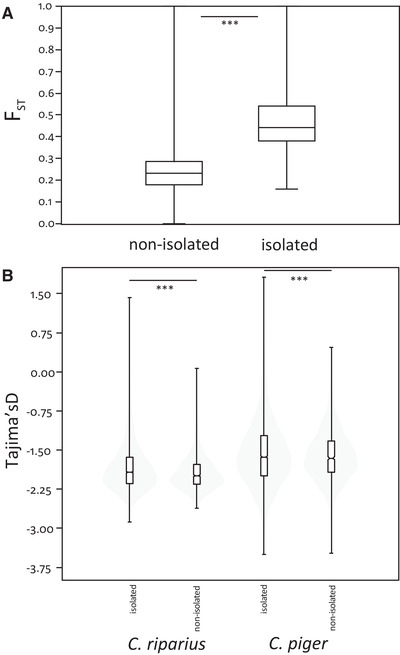
Comparisons of nonisolated versus isolated parts of the genome. (A) Boxplot of population differentiation among nonisolated and isolated 10‐kb windows measured as *F*_ST_. The difference of means (0.235 and 0.477, respectively) was highly significant (*P* < 0.001, Cohen's *d* = 2.46). (B) Violin plots of Tajima's *D* for nonisolated and isolated windows for the two species, respectively. Comparisons were significantly different (*P* < 0.001).

The inferred isolated windows differed significantly from the nonisolated ones in all parameters estimated across the genome. The largest differences were found in *F*_ST_, *D_xy_*_,_ and genetic diversity (*θ*), all having large effect sizes (Figs. 3B, S9B, and S9C). LD as a proxy for recombination rate only had a small effect (Supporting Information 1.5; Fig. S9A).

Observed isolated regions were with on average 30.2 ± 32.8 kb significantly larger than expected by chance (simulated mean 13.3 ± 0.1 kb, *P* < 0.0001, Supporting Information 1.5). The isolated regions contained 6723 out of 13,449 annotated genes, which is almost twice the expected number, assuming an even distribution of genes across the genome (*χ*² = 2957, *P* < 0.0001). The genes in isolated regions were significantly enriched for GO terms associated with protein synthesis, ranging from transcription to protein modification in the category *Biological Function* (Table 1). In the category *Cellular Component*, mainly GO terms relating to complex cell structures were found to be enriched, whose function requires close molecular interaction of many constituting protein or RNA components, such as ribosome, mitochondrion and nucleus (Table 1).

**Table 1:** Gene ontology (GO) term enrichment analysis in isolated parts of the genome. Significantly (*P* < 0.05) overrepresented GO terms in the categories biological process (BP) and cellular component (CC) for isolated regions. The column “Annotated” shows the number of genes annotated with the respective GO term, the column “Observed” the number of these genes in the isolated region, and the column “Expected” the number expected if these genes were equally distributed over the entire genome. Probability values for the number of observed genes being within random expectations are based on the weight Fisher algorithm

Category	GO ID	Term	Annotated	Observed	Expected	*P*‐value
BP	0006412	Translation	189	150	101.06	1.50 × 10^–9^
BP	0006468	Protein phosphorylation	215	149	114.96	2.70 × 10^–7^
BP	0006886	Intracellular protein transport	86	65	45.98	3.30 × 10^–5^
BP	0016192	Vesicle‐mediated transport	86	63	45.98	0.00054
BP	0006457	Protein folding	45	35	24.06	0.00063
BP	0006511	Ubiquitin‐dependent protein catabolic process	52	39	27.8	0.00088
BP	0006260	DNA replication	48	40	25.67	0.00095
BP	0006414	Translational elongation	15	13	8.02	0.00189
BP	0006396	RNA processing	145	106	77.53	0.002
BP	0051604	Protein maturation	20	16	10.69	0.00353
BP	0006413	Translational initiation	13	12	6.95	0.00356
BP	0006418	tRNA aminoacylation for protein translation	44	32	23.53	0.00688
BP	0045454	Cell redox homeostasis	41	30	21.92	0.00764
BP	0000413	Protein peptidyl‐prolyl isomerization	15	13	8.02	0.00773
BP	0006357	Regulation of transcription by RNA polymerase II	31	24	16.58	0.0084
BP	0018205	Peptidyl‐lysine modification	22	20	11.76	0.01226
BP	0006364	rRNA processing	20	15	10.69	0.01272
BP	0006281	DNA repair	59	41	31.55	0.01635
BP	0051276	Chromosome organization	74	52	39.57	0.02272
BP	0034968	Histone lysine methylation	6	6	3.21	0.0233
BP	0050794	Regulation of cellular process	813	437	434.71	0.02332
BP	0006351	Transcription, DNA‐templated	386	197	206.39	0.02868
BP	0046128	Purine ribonucleoside metabolic process	9	8	4.81	0.03142
BP	0006270	DNA replication initiation	9	8	4.81	0.03142
BP	0016311	Dephosphorylation	46	26	24.6	0.03362
BP	0072594	Establishment of protein localization to organelle.	20	15	10.69	0.04344
BP	0006090	Pyruvate metabolic process	15	11	8.02	0.04356
BP	0031047	Gene silencing by RNA	5	5	2.67	0.04362
BP	0040029	Regulation of gene expression, epigenetics	5	5	2.67	0.04362
BP	0007271	Synaptic transmission, cholinergic	5	5	2.67	0.04362
BP	0002098	tRNA wobble uridine modification	5	5	2.67	0.04362
BP	0051049	Regulation of transport	5	5	2.67	0.04362
BP	0042454	Ribonucleoside catabolic process	5	5	2.67	0.04362
BP	0031123	RNA 3'‐end processing	5	5	2.67	0.04362
BP	0072523	Purine‐containing compound catabolic process	5	5	2.67	0.04362
BP	0050906	Detection of stimulus involved in sensory perception	5	5	2.67	0.04362
CC	0005840	Ribosome	114	92	65.65	1.30 × 10^–6^
CC	0032991	Protein‐containing complex	633	441	364.53	3.40 × 10^–5^
CC	0005634	Nucleus	472	323	271.81	4.00 × 10^–5^
CC	0044444	Cytoplasmic part	387	286	222.86	0.0022
CC	0005739	Mitochondrion	83	65	47.8	0.0122
CC	0005852	Eukaryotic translation initiation factor 3 complex	12	11	6.91	0.0129
CC	0019773	Proteasome core complex, alpha‐subunit complex	7	7	4.03	0.0209
CC	0005789	Endoplasmic reticulum membrane	31	25	17.85	0.0226
CC	0005743	Mitochondrial inner membrane	37	28	21.31	0.026
CC	0044424	Intracellular part	1151	792	662.83	0.0286
CC	0090575	RNA polymerase II transcription factor complex	13	11	7.49	0.0401
CC	0044428	Nuclear part	110	83	63.35	0.0486
CC	0000786	Nucleosome	58	40	33.4	0.049

### TEMPORAL DIVERGENCE DYNAMICS

The mean divergence time for isolated 10‐kb windows was estimated to 4,598,663 ± 1,588,340 generations ago, corresponding to 3.06 ± 1.05 *N*_e_ generations, using a molecular clock approach. The frequency distribution of estimated divergence times was comparatively centered and deviated significantly from normality (Shapiro‐Wilk's *W* = 0.99, *P* < 0.0001; Fig. 1B). The ABC inference framework identified the *episodic* model (Fig. 1B) as producing most similar results compared to the empirical data, indicated by a Bayes factor of 1.

A linear model (LM; see Supporting Information 1.5) showed that the length of isolated regions was significantly influenced by strength of selection (LM: *F* = 169.92, *P* < 0.0001, *η* ^2^ = 0.091), divergence time (LM: *F* = 117.97, *P* < 0.0001, *η* ^2^ = 0.063, Fig. 1C) and LD (LM: F = 21.97, *P* < 0.0001, *η* ^2^ = 0.012) as well as all possible two‐way interactions (LM selection:divergence time: *F* = 10.68, *P* = 0.0011, *η* ^2^ = 0.006; LM divergence time:LD: *F* = 6.63, *P* = 0.01, *η* ^2^ = 0.004; LM selection:LD: *F* = 3.87, *P* = 0.049, *η* ^2^ = 0.002).

### NEGATIVE TD THROUGHOUT THE GENOME

TD was skewed toward negative values in both isolated and nonisolated windows. In nonisolated windows, values ranged between 0.064 and −2.611 in *C. riparius* and between 0.47 and −3.48 in *C. piger* (Fig. 3B). The 5th and 95th percentiles were −1.39, −2.36, and −0.79, −2.32, respectively. A total of 365 isolated windows in *C. riparius* and 464 in *C. piger* were below the lower threshold. Of these isolated windows, 68 occurred in both taxa, which was significantly more than expected by chance (randomization test [Pfenninger et al. 2015]: mean expected = 31.5, *P* < 0.001). The upper expectation threshold for TD was exceeded by 297 isolated windows in *C. riparius* and 396 in *C. piger*. The overlap of 134 windows was much higher than expected by chance (randomization test [Pfenninger et al. 2015]: mean expected = 22, *P* < 0.001). A list of genes in the windows considered to be driven by nonneutral processes (see methods above) can be found in Supporting Information 3.

### SELECTION ON PROTEIN CODING GENES IN ISOLATED REGIONS

For the 6723 genes in the isolated regions, McDonald‐Kreitman tests showed a significant accumulation of either synonymous or nonsynonymous divergent SNPs for 451 genes. Neutrality index indicated that 421 were under negative selection, whereas 30 showed signs of positive selection. No significantly enriched GO terms were found among the putatively positive selected genes. From the 392 fixed divergent nonsynonymous SNPs in positively selected genes, a proportion of 0.89 was estimated to have become fixed by this process. The list of positively selected genes in isolated regions can be found in Supporting Information 3.

## Discussion

In this study, we focused on evolution and dynamics of the divergence landscape in the face of gene flow between two sister species of nonbiting midges, *C. riparius* and *C. piger*. We used a novel PCA approach not requiring admixture‐free reference individuals to unravel the heterogeneous genomic differentiation across the genome. We were able to distinguish regions of significantly reduced gene flow (“isolated”) from apparently freely exchangeable ones (“nonisolated”). We analyzed the driving forces behind formation of islands of divergence, inferred the temporal divergence dynamics, and investigated whether the divergence processes are still ongoing or if a stable steady‐state selection‐migration‐drift equilibrium was reached.

### METHODOLOGICAL CONSIDERATIONS

Sampling five different *C. riparius* populations and comparing them to a single *C. piger* population to derive taxa‐wide genomic differentiation patterns might have been problematic; however, this approach proved to be sufficient. The equidistance between the respective *C. piger* and all *C. riparius* populations in the PCA (Fig. 2A) showed that the specific differences are rather categorical and less dependent on geographical proximity, local adaptation, or differential introgression. The homogeneous, specific differences may result from homogenizing intraspecific gene flow (Kumar et al. 2017), leading to similar isolation patterns across the taxa's range, even in populations never in direct contact with the sister taxon. High intraspecific gene flow among *C. riparius* populations (Waldvogel et al. 2018) coupled with low hybridization rates between the taxa (Pedrosa et al. 2017; Foucault et al. 2019) supports this explanation.

We could reliably identify the heterogeneous divergence landscape among the recurrently hybridizing taxa. To identify mutually isolated and therefore highly differentiated genomic regions between hybridizing taxa, most studies use summary statistics such as *F*_ST_ or similar (Nosil et al. 2009; Wolf and Ellegren 2017). However, these statistics are criticized for losing explanatory power due to multiple factors such as selection, recombination, and mutation rate, which all vary across genomes (Hodgkinson and Eyre‐Walker 2011; Ravinet et al. 2017). This is particularly true when using whole genome data at late stages of divergence (Meirmans and Hedrick 2011) as these effects accumulate with time and number of loci considered.

Alternatively, ancestry of genome blocks and thus regions of introgression and isolation can be inferred on the basis of (putatively) nonadmixed reference individuals (Price et al. 2009; Schaefer et al. 2017). However, these individuals probably do not exist in many cases for a variety of reasons, for example, recurrent hybridization since initial divergence as in the current case. Moreover, even in populations never in direct contact with the sister lineage, genomic integrity may be compromised by intraspecific gene flow (Kumar et al. 2017). Therefore, the basic assumption of reference‐based admixture analysis is likely unwittingly violated in many instances.

By maximizing the variance among correlated multivariate variables, a SNP‐based PCA naturally finds on its first axis the SNPs contributing most to the differences between the highest order taxa. If these SNPs are spatially clustered and not homogeneously distributed across the genome, it is reasonable to assume that gene flow rates differ locally along the genome among the inferred taxa. Due to the time necessary for a given region to accumulate a certain number of divergent mutations, however, the method has natural limits. Simulations showed that for the most recent 2.5 million (1.7 *N*_e_) generations, likely not all isolated windows were identified as such, thus underestimating the number and size of isolated regions for this period (false negative rate, Fig. S7A). On the other hand, windows flagged as isolated had a very low false positive rate even for relatively recently isolated windows (>200,000 generations; Fig. S7B).

As expected, identified isolated and nonisolated windows differed significantly in net divergence (Figs. 3A and S9C). Additionally, we found a strong positive correlation between the identified isolated windows and *F*_ST_ values. This suggests that an *F*_ST_ outlier approach (Nosil et al. 2009; Wolf and Ellegren 2017) would have resulted in a comparable inference of the heterogeneous divergence landscape. The remaining differences could be explained by *F*_ST_ evaluating relative allele frequency differences, whereas the PCA approach assesses reduction in gene flow as well as divergent mutation accumulation in certain genomic regions.

As in all window approaches, the spatial resolution for delimiting isolated genomic regions is influenced by the chosen window size. It was necessary to find a balance between statistical power, which increases with increasing window size, and a biologically relevant resolution, which depends on the local recombination rate (Teo et al. 2009). Using LD as a proxy for the recombination rate showed that average haplotype length in *C. riparius* was significantly smaller than the chosen 10‐kb windows (Schmidt et al. 2020). Consequently, some windows flagged as either isolated or nonisolated may actually represent a patchwork of both. This renders the estimation of all parameters calculated based on windows less accurate. However, choosing a much smaller window size would have made several parameter estimations impossible, due to the lack of informative sites, and increased variance of estimates. Choosing a suitable window size therefore strongly depends on the system under investigation and cannot be generalized here.

### EXTENT OF GI

Mutually isolated regions between *C. riparius* and *C. piger* were dispersed across almost two thirds of all scaffolds, but accounted for only 28% of the total assembled genome. Interestingly, however, the isolated regions contained half of all annotated genes. Conversely, that means that 72% of the genome, containing the remaining 50% of genes, appears to be freely exchangeable between the taxa. Therefore, the nonisolated parts, comprising the majority of the genome, resemble a shared gene pool, which is reflected in higher *N*_e_ (represented by *θ*) compared to isolated regions (Fig. S9B). Species identity hence seems to be based on only half of the genes.

Acknowledging the restrictions and limitations of GO annotations and their interpretations (Sangar et al. 2007), we found conspicuously many genes in isolated regions associated with processes or structures that require close molecular interactions with protein or RNA coding genes. These included genes associated with mitochondrial functions, protein synthesis at ribosomes or chromosome organization, and replication in the nucleus. Selection for efficiency in processes requiring close molecular or functional interaction of several genomic regions (e.g., oxidative phosphorylation in eukaryotes; Zhang and Broughton 2013) could be the reason. Disruption of these interactions has been shown to reduce hybrid fitness (Ellison et al. 2008; Zhang and Broughton 2013) and thereby foster divergence. It is therefore not surprising that strong postzygotic RI barriers exist (Hägele 1984, 1999; Armitage et al. 1995), leading to reduced fitness in all (back)crossing directions (Foucault et al. 2019). In particular, the divergent genes associated with chromosome organization and replication could account for observed chromosome aberrations in hybrids (Hägele 1984).

Other prime candidates often involved in divergence are sex‐linked genes (Qvarnström and Bailey 2009), which fits the observation that the sex determining region (SDR; Supporting Information 1.3) was isolated and even showed above average *D_xy_* for isolated regions Also, the enriched GO term *detection of stimulus involved in sensory perception* in isolated regions is in accordance to the observation that genes in possible relation to intraspecific communication play a disproportionately large role in divergence (Harr 2006; Lawniczak et al. 2010; Nadeau et al. 2012).

### DYNAMICS OF THE DIVERGENCE PROCESS

Temporal divergence dynamics has been mostly inferred from multiple species or population pairs in different stages of divergence (Martin et al. 2013; Burri et al. 2015; Riesch et al. 2017). We analyzed the divergence dynamic from a single pair of taxa by using a molecular clock approach. Once gene flow and thus subsequent recombination is suppressed by selection against introgression, the affected genome regions should start to accumulate mutations in each of the divergent lineages independently. Knowing the mutation rate (Oppold and Pfenninger 2017), it was therefore possible to estimate the approximate time of divergence for each isolated region. Even though the variance of molecular clock estimates is large (Kumar and Blair Hedges 2016) and mutation rates (Hodgkinson and Eyre‐Walker 2011), recombination rates (Nachman 2002) as well as selection are probably not constant over the entire genome, the distribution of estimates should nevertheless entail information on the temporal dynamics of divergence.

We used a coalescent approach to simulate expected divergence time distributions for five possible divergence scenarios and compared them with the empirically derived divergence time estimates. Applying an ABC inference framework, our approach shares the characteristic of all model selection approaches that it can only distinguish between the models tested, which may or may not include the true one (Johnson and Omland 2004).

The empirical data showed highest similarity with the episodic model, which assumed a short divergence period in the past (Fig. 1B). Calculating divergence time per window suggested that most of the isolated windows emerged during a relatively short time span about 4.6 million (3 N_e_) generations ago. Assuming eight to 10 generations per year on average, this would place the divergence into the Mid‐Pleistocene as a rough estimate. As the expected mean time (as well as the standard deviation) to coalescence of two alleles in a diploid population is 2 N_e_ (Nei and Takahata 1993), we expect that lineage sorting among isolated loci is complete for the majority, but certainly not for all loci. This showed that the divergence process among the sister species is quite advanced and not recent. Such a scenario, reminding of a “punctuated equilibrium” as described by Eldredge and Gould (1972), in the case of divergence with gene flow is supported by theory: Simulations by Flaxman et al. (2014) have shown that initially gradual adaptive change can generate nonlinear transitions causing rapid emergence of complete RI, resulting in distinct bursts of speciation. Although our findings suggested that the divergence process conformed this model, it did not result in complete RI. It remained, however, unclear what triggered the divergence. An allopatric phase with differential ecological adaptation followed by secondary contact cannot be excluded. Another, nonexclusive explanation is that the beginning expansion of the *Cla‐element* caused reduced gene flow by intragenomic incompatibilities (Oppold et al. 2017). However, our data did not suggest a close spatial association between large *Cla* clusters (or other repetitive elements) and divergence islands.

Factors influencing the length of isolated genome regions were further examined using a LM with selection, recombination, and divergence time as independent variables, as suggested by the Feder et al. model (2012b). Contrary to expectations (Martin et al. 2019), the recombination rate proxy explained only about 1% of length distribution variance. This seemed contradictory to the isolation of scaffolds known to contain the SDR and thus a region of known reduced recombination. However, reduced recombination may have simply contributed to maintain isolation of divergently selected sex‐linked genes. Selection had the biggest impact, explaining about 9% of variance, followed by divergence time (6%). Albeit significant, all interactions between the variables had minor influence (<1%). The model as a whole explained only about 18%. This may have several, nonexclusive reasons. First, *θ* is known to be unbiased under neutrality and constant population growth (population growth rate constantly >1 through time) only (Subramanian 2016). These assumptions are probably not met in multivoltine taxa under a multivariate, fluctuating selection regime that undergo seasonal boom‐bust cycles (Pfenninger and Foucault 2020). Also, LD and divergence time estimates lose accuracy under nonneutral conditions (Kumar 2005; Slatkin 2008). In addition, DH and GH may inherently lead to a heterogeneous differentiation pattern in divergence with gene flow scenarios (Flaxman et al. 2013; Wolf and Ellegren 2017).

Likewise, pervasive selection by adaptive tracking in conjunction with the seasonally fluctuating population size in both species with an exponential increase over several generations in summer with a setback during a single generation in winter (Pfenninger and Foucault 2020) may explain the strongly skewed TD distribution toward negative values both in isolated and nonisolated regions. Simulations have shown that demographic fluctuations alone can produce such a pattern (Fig. S8), which would be amplified by constant selection (Excoffier et al. 2009). This bias made traces of divergently selected sites, typical for DH (Via 2012), harder to detect in the site frequency spectrum because they require more extreme negative TD values. Hence, our analysis might underestimate the portion of isolated windows showing signs of recent divergent selection (7% and 9% in *C. riparius* and *C. piger*, respectively). Other mutually nonexclusive explanations are (i) the majority of divergent selective sweeps occurred so long ago that most of the isolated regions are back in mutation‐drift equilibrium or (ii) the sweeps were soft and thus did not produce a strong signal. Against this overall trend, a substantial fraction of isolated windows in both species, with a highly significant overlap, showed a lack of rare alleles (TD > 0 and exceeding the upper expectation threshold), indicating balancing selection. Both may be linked to multivoltinity of the species in which successive generations experience very different selection regimes throughout the seasons and over time (Foucault et al. 2019; Pfenninger and Foucault 2020).

MKT results indicated that most of the genes within isolated regions were not affected by (divergent) protein evolution. Thus, most of the genes within divergent regions either hitchhiked with the positively selected genes, which is most likely given the high gene density of the *Chironomus* genome, and/or hitchhiked with selection acting on expression control in noncoding parts.

Overall, parts of the isolated regions (still) showed signs of divergent and/or positive selection that fostered divergence. However, the isolation likely is kept up for a larger portion by background selection. Only a very small fraction of isolated windows (1.3%) showed signs of recent divergent selection in both species. It appears that the taxa are in a stable steady‐state selection‐migration‐drift equilibrium corresponding to phase 2 (DH) of the four‐phase model of speciation with gene flow (Feder et al. 2012b, 2013). Similarly, Burri et al. (2015) found linked selection to lead to the formation of heterogeneous divergence landscapes in diverging *Ficedula* flycatcher species. But at the same time, they identified local recombination rate to have a strong effect on the build‐up of isolation throughout the genome and not only in regions with known reduced recombination like, for example, the SDR in *Chironomus*. Avian species are known for their particularly variable recombination rate suggesting that its influence on isolation may be considered taxon specific rather than general (Kawakami et al. 2014). Similar to our results, studies on stick insects (Riesch et al. 2017) and flycatchers (Burri et al. 2015) question the concept of constantly growing isolation regions during the divergence process inevitably reaching complete RI, contrasting to theoretical expectations (Wu 2001; Feder et al. 2012b). It seems that, despite incomplete RI, species identity in these cases is maintained by other pre‐ and postzygotic reproductive barriers such as ecological selection (Schwenk et al. 2000), behavioral differences (Canestrelli et al. 2017), or genetic incompatibilities (Foucault et al. 2019).

## CONCLUSION

The steadily accumulating evidence for speciation with gene flow reaching a stable intermediate steady state suggests the widespread occurrence of selection‐migration‐drift equilibria. Closely related taxa may thus share significant parts of their genomes over extended evolutionary times while retaining their specific identity.

## AUTHOR CONTRIBUTIONS

MP conceived the study. DS performed sequencing analyses. DS and MP analyzed data and drafted the manuscript together. 

## DATA ARCHIVING

Trimmed whole genome individual resequencing data available at European Nucleotide Archive (ENA) project number: PRJEB35704. Sequence data obtained from Waldvogel et al. (2018, see Supporting Information 1.1) available at European Nucleotide Archive (ENA) project number: PRJEB24868.

## 1 CONFLICT OF INTEREST

The authors declare no conflict of interest.

Associate Editor: Z. Gompert

## Supporting information

Figure S1: Map of Europe displaying sample sites: MG: Hesse, Germany (50.188297, 9.214170); NMF: Lorraine, France (49.008298, 6.223934); MF: Lyon, France (46.017340, 4.911689); SI: Piemont, Italy (45.248064, 7.610818); SS: Andalusia, Spain (37.807905, –4.765055).
Figure S2: Cumulative factor score sum of SNPs associated with (species splitting) PC1 in the PCA used to identify the isolated windows (see Fig. 2a).
Figure S3: LD decay curve of maximum likelihood estimates of r2.
Figure S4: Experimental design for coalescence simulations.
Figure S5: Maximum likelihood estimation of individual ancestry based on 900,345 SNPs (quality filtered, LD‐pruned) using ADMIXTURE version 1.3.0 with *K* = 2.
Figure S6: Principal component analysis of the LD‐pruned SNP dataset.
Figure S7: Inference of lower temporal resolution limit with coalescence simulations.
Figure S8: Influence of fluctuating population size on distribution of Tajima's *D*.
Figure S9: Comparisons of nonisolated versus isolated parts of the genome in view of factors influencing the length of isolated regions.
Table S1: Barcoding results for all 36 samples based on universal mitochondrial COI (Folmer et al. 1994), taxa‐specific nuclear L44 (Oppold et al. 2016) as well as microsatellites markers.
Table S2: Gene ontology (GO) term enrichment analysis of positively selected genes found in isolated parts of the genome.
Table S3: Mean values of all four summary statistics per divergence scenario describing the divergence time distributions used in the ABC model inference approach as well as the empirical data.
Table S4: Confusion matrix based on leave‐on‐out cross validation for 100 samples for each of the five models, applying a tolerance rate of 0.01 and using the “rejection” method.

Supplementary Material

Supplementary Material

## References

[evl3204-bib-0001] Alexa, A. , and J. Rahnenführer . 2018 topGO: enrichment analysis for gene ontology. R package version 2.40.0.

[evl3204-bib-0002] Alexander, D. H. , J. Novembre , and K. Lange . 2009 Fast model‐based estimation of ancestry in unrelated individuals. Genome Res. 19:1655–1664. 19648217 10.1101/gr.094052.109 PMC2752134

[evl3204-bib-0003] Armitage, P. D. , P. S. Cranston , and L. C. V. Pinder . 1995 The Chironomidae: biology and ecology of non‐biting midges. Springer Science+Business Media, Dordrecht, the Netherlands.

[evl3204-bib-0004] Baran, Y. , I. Quintela , Á. Carracedo , B. Pasaniuc , and E. Halperin . 2013 Enhanced localization of genetic samples through linkage‐disequilibrium correction. Am. J. Hum. Genet. 92:882–894. 23726367 10.1016/j.ajhg.2013.04.023 PMC3675263

[evl3204-bib-0005] Burri, R. , A. Nater , T. Kawakami , C. F. Mugal , P. I. Olason , L. Smeds , et al. 2015 Linked selection and recombination rate variation drive the evolution of the genomic landscape of differentiation across the speciation continuum of *Ficedula* flycatchers. Genome Res. 25:1656–1665. 26355005 10.1101/gr.196485.115 PMC4617962

[evl3204-bib-0006] Canestrelli, D. , R. Bisconti , A. Chiocchio , L. Maiorano , M. Zampiglia , and G. Nascetti . 2017 Climate change promotes hybridisation between deeply divergent species. PeerJ 2017:1–16. 10.7717/peerj.3072 PMC5366042 28348926

[evl3204-bib-0007] Chen, S. Y. , T. Tsubouchi , B. Rockmill , J. S. Sandler , D. R. Richards , G. Vader , et al. 2008 Global analysis of the meiotic crossover landscape. Dev. Cell 15:401–415. 18691940 10.1016/j.devcel.2008.07.006 PMC2628562

[evl3204-bib-0008] Danecek, P. , A. Auton , G. Abecasis , C. A. Albers , E. Banks , M. A. DePristo , et al. 2011 The variant call format and VCFtools. Bioinformatics 27:2156–2158. 21653522 10.1093/bioinformatics/btr330 PMC3137218

[evl3204-bib-0009] DePristo M. A. , E. Banks , R. Poplin , K. V. Garimella , J. R. Maguire , C. Hartl , et al. 2011 A framework for variation discovery and genotyping using next‐ generation DNA sequencing data. Nat Genet 43:491–498. 21478889 10.1038/ng.806 PMC3083463

[evl3204-bib-0010] Duranton, M. , N. Bierne , F. Bonhomme , P.‐A. Gagnaire , C. Fraïsse , and F. Allal . 2018 The origin and remolding of genomic islands of differentiation in the European sea bass. Nat. Commun. 9:1–11. 29955054 10.1038/s41467-018-04963-6 PMC6023918

[evl3204-bib-0011] Eldredge, N. , and S. J. Gould . 1972 Punctuated equilibria: an alternative to phyletic gradualism Pp. 82–115 *in* T. J. M. Schopf , ed. Essential readings in evolutionary biology. Cooper & Co, San Francisco, CA.

[evl3204-bib-0012] Ellegren, H. , L. Smeds , R. Burri , P. I. Olason , N. Backström , T. Kawakami , et al. 2012 The genomic landscape of species divergence in *Ficedula* flycatchers. Nature 491:756–760. 23103876 10.1038/nature11584

[evl3204-bib-0013] Ellison, C. K. , O. Niehuis , and J. Gadau . 2008 Hybrid breakdown and mitochondrial dysfunction in hybrids of *Nasonia* parasitoid wasps. J. Evol. Biol. 21:1844–1851. 18811665 10.1111/j.1420-9101.2008.01608.x

[evl3204-bib-0014] Excoffier, L. 2000 Computer note. SIMCOAL: a general coalescent program for the simulation of molecular data in interconnected populations with arbitrary demography. J. Hered. 91:506–509. 11218093 10.1093/jhered/91.6.506

[evl3204-bib-0015] Excoffier, L. , M. Foll , and R. J. Petit . 2009 Genetic consequences of range expansions. Annu. Rev. Ecol. Evol. Syst. 40:481–501.

[evl3204-bib-0016] Faure, B. , D. Jollivet , A. Tanguy , F. Bonhomme , and N. Bierne . 2009 Speciation in the deep sea: multi‐locus analysis of divergence and gene flow between two hybridizing species of hydrothermal vent mussels. PLoS One 4:1–15. 10.1371/journal.pone.0006485 PMC2715857 19649261

[evl3204-bib-0017] Feder, A. F. , D. A. Petrov , and A. O. Bergland . 2012a LDx: estimation of linkage disequilibrium from high‐throughput pooled resequencing data. PLoS One 7:1–7. 10.1371/journal.pone.0048588 PMC3494690 23152785

[evl3204-bib-0018] Feder, J. L. , S. P. Egan , and P. Nosil . 2012b The genomics of speciation‐with‐gene‐flow. Trends Genet. 28:342–350. 22520730 10.1016/j.tig.2012.03.009

[evl3204-bib-0019] Feder, J. L. , S. M. Flaxman , S. P. Egan , and P. Nosil . 2013 Hybridization and the build‐up of genomic divergence during speciation. J. Evol. Biol. 26:261–266. 23324002 10.1111/jeb.12009

[evl3204-bib-0020] Feulner, P. G. D. , F. ééJ J. Chain , M. Panchal , Y. Huang , C. Eizaguirre , M. Kalbe , et al. 2015 Genomics of divergence along a continuum of parapatric population differentiation. PLoS Genet. 11:1–18. 10.1371/journal.pgen.1004966 PMC4334544 25679225

[evl3204-bib-0021] Flaxman, S. M. , J. L. Feder , and P. Nosil . 2013 Genetic hitchhiking and the dynamic buildup of genomic divergence during speciation with gene flow. Evolution. 67:2577–2591. 24033168 10.1111/evo.12055

[evl3204-bib-0022] Flaxman, S. M. , A. C. Wacholder , J. L. Feder , and P. Nosil . 2014 Theoretical models of the influence of genomic architecture on the dynamics of speciation. Mol. Ecol. 23:4074–4088. 24724861 10.1111/mec.12750

[evl3204-bib-0023] Foucault, Q. , A. Wieser , C. Heumann‐Kiesler , J. Diogo , B. Cocchiararo , C. Nowak , et al. 2019 An experimental assessment of reproductive isolation and its consequences for seasonal hybridization dynamics. Biol. J. Linn. Soc. 126:327–337.

[evl3204-bib-0024] Gunderina, L. I. , and E. A. Salina . 2003 Polymorphism and divergence of multilocus DNA markers in sibling species *Chironomus riparius* Meigen and *Chironomus piger* Strenzke (Diptera, Chironomidae). Russ. J. Genet. 39:890–895. 14515462

[evl3204-bib-0025] Haas, H. , and K. Strenzke . 1957 Experimentelle Untersuchungen über den Einfluß der ionalen Zusammensetzung des Mediums auf die Entwicklung der Analpapillen von *Chironomus thummi* . Biol. Zent. Bl. 76:513–528.

[evl3204-bib-0026] Hägele, K. 1984 Different hybrid effects in reciprocal crosses between *Chironomus thummi thummi* and *Ch. th. piger* including spontaneous chromsome aberrations and sterility. Genetica 63:105–111.

[evl3204-bib-0027] Hägele, K. 1999 Hybrid syndrome‐induced postzygotic reproductive isolation: a second reproduction barrier in *Chironomus thummi* (Diptera, Chironomidae). J. Zool. Syst. Evol. Res. 37:161–164.

[evl3204-bib-0028] Harr, B. 2006 Genomic islands of differentiation between house mouse subspecies. Genome Res. 16:730–737. 16687734 10.1101/gr.5045006 PMC1473184

[evl3204-bib-0029] Hodgkinson, A. , and A. Eyre‐Walker . 2011 Variation in the mutation rate across mammalian genomes. Nat. Rev. Genet. 12:756–766. 21969038 10.1038/nrg3098

[evl3204-bib-0030] Johnson, J. B. , and K. S. Omland . 2004 Model selection in ecology and evolution. Trends Ecol. Evol. 19:101–108. 16701236 10.1016/j.tree.2003.10.013

[evl3204-bib-0031] Kassambara, A. , and F. Mundt . 2017 factoextra: extract and visualize the results of multivariate data analysis. R package version 1.0.7.

[evl3204-bib-0032] Kawakami, T. , L. Smeds , N. Backström , A. Husby , A. Qvarnström , C. F. Mugal , et al. 2014 A high‐density linkage map enables a second‐generation collared flycatcher genome assembly and reveals the patterns of avian recombination rate variation and chromosomal evolution. Mol. Ecol. 23:4035–4058. 24863701 10.1111/mec.12810 PMC4149781

[evl3204-bib-0033] Keyl, H.‐G. 1965 A demonstrable local and geometric increase in the chromosomal DNA of *Chironomus* . Experientia 21:191–193. 5844173 10.1007/BF02141878

[evl3204-bib-0034] Kiknadze, I. I. , A. I. Shilova , I. E. Kerkis , N. A. Shobanov , N. I. Zelentsov , L. P. Grebenjuk , et al. 1991 Kariotipy i morfologiya lichinok triby Chironomini: atlas. (Larvae Karyotypes and Morphology in the Tribe Chironomini: atlas). Nauka, Novosibirsk, Russia.

[evl3204-bib-0035] Kumar, S. 2005 Molecular clocks: four decades of evolution. Nat. Rev. Genet. 6:654–662. 16136655 10.1038/nrg1659

[evl3204-bib-0036] Kumar, S. , and S. Blair Hedges . 2016 Advances in time estimation methods for molecular data. Mol. Biol. Evol. 33:863–869. 26882983 10.1093/molbev/msw026 PMC5870647

[evl3204-bib-0037] Kumar, S. , G. Stecher , M. Li , C. Knyaz , and K. Tamura . 2018 MEGA X: molecular evolutionary genetics analysis across computing platforms. Mol. Biol. Evol. 35:1547–1549. 29722887 10.1093/molbev/msy096 PMC5967553

[evl3204-bib-0038] Kumar, V. , F. Lammers , T. Bidon , M. Pfenninger , L. Kolter , M. A. Nilsson , et al. 2017 The evolutionary history of bears is characterized by gene flow across species. Sci. Rep. 7:1–10. 28422140 10.1038/srep46487 PMC5395953

[evl3204-bib-0039] Lawniczak, M. K. N. , S. J. Emrich , A. K. Holloway , A. P. Regier , M. Olson , B. White , S. Redmond , et al. 2010 Widespread divergence between incipient *Anopheles gambia* species revealed by whole genome sequences. Science 330:512–514. 20966253 10.1126/science.1195755 PMC3674514

[evl3204-bib-0040] Li, H. 2011 A statistical framework for SNP calling, mutation discovery, association mapping and population genetical parameter estimation from sequencing data. Bioinformatics 27:2987–2993. 21903627 10.1093/bioinformatics/btr509 PMC3198575

[evl3204-bib-0041] Martin, S. H. , K. K. Dasmahapatra , N. J. Nadeau , C. Salazar , J. R. Walters , F. Simpson , et al. 2013 Genome‐wide evidence for speciation with gene flow in *Heliconius* butterflies. Genome Res. 23:1817–1828. 24045163 10.1101/gr.159426.113 PMC3814882

[evl3204-bib-0042] Martin, S. H. , J. W. Davey , C. Salazar , and C. D. Jiggins . 2019 Recombination rate variation shapes barriers to introgression across butterfly genomes. PLoS Biol. 17:1–28. 10.1371/journal.pbio.2006288 PMC6366726 30730876

[evl3204-bib-0043] Meirmans, P. G. , and P. W. Hedrick . 2011 Assessing population structure: FSTand related measures. Mol. Ecol. Resour. 11:5–18. 21429096 10.1111/j.1755-0998.2010.02927.x

[evl3204-bib-0044] Miehlbradt, J. , and D. Neumann . 1976 Reproduktive isolation durch optische Schwarmmarken bei den sympatrischen *Chironomus thummi* und *Ch. piger* . Behaviour 58:272–297.

[evl3204-bib-0045] Nachman, M. W. 2002 Variation in recombination rate across the genome: evidence and implications. Curr. Opin. Genet. Dev. 12:657–663. 12433578 10.1016/s0959-437x(02)00358-1

[evl3204-bib-0046] Nadeau, N. J. , A. Whibley , R. T. Jones , J. W. Davey , K. K. Dasmahapatra , S. W. Baxter , et al. 2012 Genomic islands of divergence in hybridizing *Heliconius* butterflies identified by large‐scale targeted sequencing. Philos. Trans. R. Soc. B Biol. Sci. 367:343–353. 10.1098/rstb.2011.0198 PMC3233711 22201164

[evl3204-bib-0047] Nei, M. , and N. Takahata . 1993 Effective population size, genetic diversity, and coalescence time in subdivided population. J. Mol. Evol. 37:240–244. 8230248 10.1007/BF00175500

[evl3204-bib-0048] Neumann, D. , M. Kramer , I. Raschke , and B. Gräfe . 2001 Detrimental effects of nitrite on the development of benthic *Chironomus* larvae, in relation to their settlement in muddy sediments. Arch. fuer Hydrobiol. 153:103–128.

[evl3204-bib-0049] Nosil, P. , D. J. Funk , and D. Ortiz‐Barrientos . 2009 Divergent selection and heterogeneous genomic divergence. Mol. Ecol. 18:375–402. 19143936 10.1111/j.1365-294X.2008.03946.x

[evl3204-bib-0050] Oppold, A. 2017 Mechanismen der genomischen Populationsdivergenz in *Chironomus riparius* entlang eines Klimagradienten. Johann Wolfgang Goethe‐Universität Frankfurt, Frankfurt, Germany.

[evl3204-bib-0051] Oppold, A.‐M. , and M. Pfenninger . 2017 Direct estimation of the spontaneous mutation rate by short‐term mutation accumulation lines in *Chironomus riparius* . Evol. Lett. 2:86–92 10.1002/evl3.8 PMC6121839 30283641

[evl3204-bib-0052] Oppold, A.‐M. , J. A. M. Pedrosa , M. Bálint , J. B. Diogo , J. Ilkova , J. L. T. Pestana , et al. 2016 Support for the evolutionary speed hypothesis from intraspecific population genetic data in the non‐biting midge *Chironomus riparius* . Proc. R. Soc. B Biol. Sci. 283:20152413. 10.1098/rspb.2015.2413 PMC4810824 26888029

[evl3204-bib-0053] Oppold, A.‐M. , H. Schmidt , M. Rose , S. L. Hellmann , F. Dolze , F. Ripp , et al. 2017 *Chironomus riparius* (Diptera) genome sequencing reveals the impact of minisatellite transposable elements on population divergence. Mol. Ecol. 26:3256–3275. 28316106 10.1111/mec.14111

[evl3204-bib-0054] Pedrosa, J. , B. Cocchiararo , T. Verdelhos , C. Nowak , A. Soares , and J. Pestana . 2017 Population genetic structure and hybridization patterns in the cryptic sister species *Chironomus riparius* and *Chironomus piger* across differentially polluted freshwater systems. Ecotoxicol. Environ. Saf. 141:280–289. 28359994 10.1016/j.ecoenv.2017.03.004

[evl3204-bib-0055] Petrova, N. A. , S. V. Zhirov , K. V. Arutiunova , and M. V. Arutiunova . 2014 On the possibility of spontaneous interspecific hybridization in the nature of representatives of sibling Species *Chironomus riparius* Kieffer and *Chironomus piger* Strenzke (Diptera, Chironomidae) from Armenia. Tsitologiia 56:170–174. 25509157

[evl3204-bib-0056] Pfeifer, B. , U. Wittelsbürger , S. E. Ramos‐Onsins , and M. J. Lercher . 2014 PopGenome: an efficient Swiss army knife for population genomic analyses in R. Mol. Biol. Evol. 31:1929–1936. 24739305 10.1093/molbev/msu136 PMC4069620

[evl3204-bib-0057] Pfenninger, M. , and C. Nowak . 2008 Reproductive isolation and ecological niche partition among larvae of the morphologically cryptic sister species *Chironomus riparius* and *C. piger* . PLoS One 3:e2157. 18478074 10.1371/journal.pone.0002157 PMC2364647

[evl3204-bib-0058] Pfenninger, M. , and Q. Foucault . 2020 Quantifying the selection regime in a natural *Chironomus riparius* population. bioRxiv 10.1101/2020.06.16.154054. 31886913

[evl3204-bib-0059] Pfenninger, M. , S. Patel , L. Arias‐Rodriguez , B. Feldmeyer , R. Riesch , and M. Plath . 2015 Unique evolutionary trajectories in repeated adaptation to hydrogen sulphide‐toxic habitats of a neotropical fish (*Poecilia mexicana*). Mol. Ecol. 24:5446–5459. 26405850 10.1111/mec.13397

[evl3204-bib-0060] Price, A. L. , A. Tandon , N. Patterson , K. C. Barnes , N. Rafaels , I. Ruczinski , et al. 2009 Sensitive detection of chromosomal segments of distinct ancestry in admixed populations. PLoS Genet. 5:e1000519. 19543370 10.1371/journal.pgen.1000519 PMC2689842

[evl3204-bib-0061] Qvarnström, A. , and R. I. Bailey . 2009 Speciation through evolution of sex‐linked genes. Heredity 102:4–15. 18781167 10.1038/hdy.2008.93

[evl3204-bib-0062] R Core Team . 2017 R: a language and environment for statistical computing. R Foundation for Statistical Computing, Vienna.

[evl3204-bib-0063] Rafajlović, M. , A. Emanuelsson , K. Johannesson , R. K. Butlin , and B. Mehlig . 2016 A universal mechanism generating clusters of differentiated loci during divergence‐with‐migration. Evolution 70:1609–1621. 27196373 10.1111/evo.12957 PMC5089645

[evl3204-bib-0064] Ravinet, M. , R. Faria , R. K. Butlin , J. Galindo , N. Bierne , M. Rafajlović , et al. 2017 Interpreting the genomic landscape of speciation: a road map for finding barriers to gene flow. J. Evol. Biol. 30:1450–1477. 28786193 10.1111/jeb.13047

[evl3204-bib-0065] Ravinet, M. , K. Yoshida , S. Shigenobu , A. Toyoda , A. Fujiyama , and J. Kitano . 2018 The genomic landscape at a late stage of stickleback speciation: high genomic divergence interspersed by small localized regions of introgression. PLoS Genet. 14:e1007358 29791436 10.1371/journal.pgen.1007358 PMC5988309

[evl3204-bib-0066] Riesch, R. , M. Muschick , D. Lindtke , R. Villoutreix , A. A. Comeault , T. E. Farkas , et al. 2017 Transitions between phases of genomic differentiation during stick‐insect speciation. Nat. Ecol. Evol. 1:82. 28812654 10.1038/s41559-017-0082

[evl3204-bib-0067] Rundle, H. D. , and P. Nosil . 2005 Ecological speciation. Ecol. Lett. 8:336–352.

[evl3204-bib-0068] Samuk, K. , B. Manzano‐Winkler , K. R. Ritz , and M. A. F. Noor . 2020 Natural selection shapes variation in genome‐wide recombination rate in *Drosophila pseudoobscura* . Curr. Biol. 30:1517–1528.e6. 32275873 10.1016/j.cub.2020.03.053

[evl3204-bib-0069] Sangar, V. , D. J. Blankenberg , N. Altman , and A. M. Lesk . 2007 Quantitative sequence‐function relationships in proteins based on gene ontology. BMC Bioinformatics 8:1–15. 17686158 10.1186/1471-2105-8-294 PMC1976327

[evl3204-bib-0070] Schaefer, N. K. , B. Shapiro , and R. E. Green . 2017 AD‐LIBS: inferring ancestry across hybrid genomes using low‐coverage sequence data. BMC Bioinformatics 18:1–22. 28376731 10.1186/s12859-017-1613-0 PMC5381037

[evl3204-bib-0071] Schmidt, E. R. 1984 Clustered and interspersed repetitive DNA sequence family of *Chironomus* . J. Mol. Biol. 178:1–15. 6090676 10.1016/0022-2836(84)90227-4

[evl3204-bib-0072] Schmidt, H. , B. Greshake , B. Feldmeyer , T. Hankeln , and M. Pfenninger . 2013 Genomic basis of ecological niche divergence among cryptic sister species of non‐biting midges. BMC Genomics 14:384. 23758757 10.1186/1471-2164-14-384 PMC3685581

[evl3204-bib-0073] Schmidt, H. , A. Waldvogel , S. L. Hellmann , T. Hankeln , and M. Pfenninger . 2020 A high‐quality genome assembly from short and long reads for the non‐biting midge *Chironomus riparius* (Diptera). G3 Genes, Genomes, Genet. 10:1151–1157. 10.1534/g3.119.400710 PMC7144091 32060047

[evl3204-bib-0074] Schwenk, K. , D. Posada , and P. D. N. Hebert . 2000 Molecular systematics of European *Hyalodaphnia*: the role of contemporary hybridization in ancient species. Proc. R. Soc. B Biol. Sci. 267:1833–1842. 10.1098/rspb.2000.1218 PMC1690753 11052533

[evl3204-bib-0075] Slatkin, M. 2008 Linkage disequilibrium–understanding the evolutionary past and mapping the medical future. Nat. Rev. Genet. 9:477–85. 18427557 10.1038/nrg2361 PMC5124487

[evl3204-bib-0076] Smith, N. G. , and A. Eyre‐Walker . 2002 Adaptive protein evolution in *Drosophila* . Nature 415:1022–1024. 11875568 10.1038/4151022a

[evl3204-bib-0077] Strenzke, K. 1957 Die systematische und ökologische Differenzierung der Gattung *Chironomus* . Analles Entomol. Fenn. 26:111–139.

[evl3204-bib-0078] Subramanian, S. 2016 The effects of sample size on population genomic analyses ‐ implications for the tests of neutrality. BMC Genomics 17:1–13. 26897757 10.1186/s12864-016-2441-8 PMC4761153

[evl3204-bib-0079] Teo, Y. Y. , A. E. Fry , K. Bhattacharya , K. S. Small , D. P. Kwiatkowski , and T. G. Clark . 2009 Genome‐wide comparisons of variation in linkage disequilibrium. Genome Res. 19:1849–1860. 19541915 10.1101/gr.092189.109 PMC2765270

[evl3204-bib-0080] Turner, T. L. , M. W. Hahn , and S. V. Nuzhdin . 2005 Genomic islands of speciation in *Anopheles gambiae* . PLoS Biol. 3:1572–1578. 10.1371/journal.pbio.0030285 PMC1182689 16076241

[evl3204-bib-0081] Via, S. 2012 Divergence hitchhiking and the spread of genomic isolation during ecological speciation‐with‐gene‐flow. Philos. Trans. R. Soc. B Biol. Sci. 367:451–460. 10.1098/rstb.2011.0260 PMC3233719 22201174

[evl3204-bib-0082] Via, S. , and J. West . 2008 The genetic mosaic suggests a new role for hitchhiking in ecological speciation. Mol. Ecol. 17:4334–4345. 18986504 10.1111/j.1365-294X.2008.03921.x

[evl3204-bib-0083] Waldvogel, A. M. , A. Wieser , T. Schell , S. Patel , H. Schmidt , T. Hankeln , et al. 2018 The genomic footprint of climate adaptation in *Chironomus riparius* . Mol. Ecol. 27:1439–1456. 29473242 10.1111/mec.14543

[evl3204-bib-0084] Weir, B. S. , and C. C. Cockerham . 1984 Estimating F‐statistics for the analysis of population structure. Evolution 38:1358–1370. 28563791 10.1111/j.1558-5646.1984.tb05657.x

[evl3204-bib-0085] Wolf, J. B. W. , and H. Ellegren . 2017 Making sense of genomic islands of differentiation in light of speciation. Nat. Rev. Genet. 18:87–100. 27840429 10.1038/nrg.2016.133

[evl3204-bib-0086] Wu, C. I. 2001 The genic view of the process of speciation. J. Evol. Biol. 14:851–865.

[evl3204-bib-0087] Yeaman, S. , and M. C. Whitlock . 2011 The genetic architecture of adaptation under migration‐selection balance. Evolution 65:1897–1911. 21729046 10.1111/j.1558-5646.2011.01269.x

[evl3204-bib-0088] Zhang, F. , and R. E. Broughton . 2013 Mitochondrial‐nuclear interactions: compensatory evolution or variable functional constraint among vertebrate oxidative phosphorylation genes? Genome Biol. Evol. 5:1781–1791. 23995460 10.1093/gbe/evt129 PMC3814189

[evl3204-bib-0089] Zheng, X. , and B. S. Weir . 2016 Eigenanalysis of SNP data with an identity by descent interpretation. Theor. Popul. Biol. 107:65–76. 26482676 10.1016/j.tpb.2015.09.004 PMC4716003

