# Supplementary material for: Genomic divergence landscape in recurrently hybridizing Chironomus sister taxa suggests stable steady state between mutual gene flow and isolation
Source: Evol Lett. 2020 Nov 6;5(1):86–100. doi: 10.1002/evl3.204 (PMC7857304; doi:10.1002/evl3.204)
Supplement: Supplementary file 1 — Figure S1: Map of Europe displaying sample sites: MG: Hesse, Germany (50.188297, 9.214170); NMF: Lorraine, France (49.008298, 6.223934); MF: Lyon, France (46.017340, 4.911689); SI: Piemont, Italy (45.248064, 7.610818); SS: Andalusia, Spain (37.807905, –4.765055). Figure S2: Cumulative factor score sum of SNPs associated with (species splitting) PC1 in the PCA used to identify the isolated windows (see Fig. 2a). Figure S3: LD decay curve of maximum likelihood estimates of r2. Figure S4: Experimental design for coalescence simulations. Figure S5: Maximum likelihood estimation of individual ancestry based on 900,345 SNPs (quality filtered, LD‐pruned) using ADMIXTURE version 1.3.0 with K = 2. Figure S6: Principal component analysis of the LD‐pruned SNP dataset. Figure S7: Inference of lower temporal resolution limit with coalescence simulations. Figure S8: Influence of fluctuating population size on distribution of Tajima's D. Figure S9: Comparisons of nonisolated versus isolated parts of the genome in view of factors influencing the length of isolated regions. Table S1: Barcoding results for all 36 samples based on universal mitochondrial COI (Folmer et al. 1994), taxa‐specific nuclear L44 (Oppold et al. 2016) as well as microsatellites markers. Table S2: Gene ontology (GO) term enrichment analysis of positively selected genes found in isolated parts of the genome. Table S3: Mean values of all four summary statistics per divergence scenario describing the divergence time distributions used in the ABC model inference approach as well as the empirical data. Table S4: Confusion matrix based on leave‐on‐out cross validation for 100 samples for each of the five models, applying a tolerance rate of 0.01 and using the “rejection” method. [file EVL3-5-86-s001.docx]

Supplementary Information 1

**Genomic divergence landscape in recurrently hybridising *Chironomus* sister taxa suggests stable steady-state between mutual gene-flow and isolation**

Dennis Schreiber^1,2^, Markus Pfenninger^1,2,3^

^1^Institute for Molecular and Organismic Evolution, Johannes Gutenberg University, Johann-Joachim-Becher-Weg 7, 55128 Mainz, Germany

^2^Dept. Molecular Ecology, Senckenberg Biodiversity and Climate Research Centre, Senckenberganlage 25, 60325 Frankfurt am Main, Germany

^3^LOEWE Centre for Translation Biodiversity Genomics (LOEWE-TBG), Senckenberganlage 25, 60325 Frankfurt am Main, Germany

# Supplementary Information

# 1.1. Sequence data processing

Overall, 36 single individual whole genome sequenced *Chironomus* specimen from 5 different sites across Europe were used for this study. Illumina reads of four *C. riparius* individuals from each of five natural populations in Europe (Suppl. Fig. 1) were obtained from Waldvogel et al. (2018, raw data available at European Nucleotide Archive (ENA) project Number: PRJEB24868). Additionally, 16 individuals (9 *C. riparius* and 7 *C. piger*) originating from a German population with known hybridisation history, sampled between June and September 2016 were sequenced (Foucault et al. 2018). Aiming at inferring the long-term effects of ongoing hybridisation on the genome, we only sequenced individuals that were not inferred as recent hybrids according to their microsatellite multilocus genotype and two barcoding loci (COI and L44 (Oppold et al. 2016; Foucault et al. 2018), Suppl. Tab. 1). Samples assigned differently by the mitochondrial data compared to the nuclear ones were assigned based on the nuclear data (microsatellites and L44). These samples were closely monitored during downstream analyses (especially during ADMIXTURE and PCA analyses (see below)). However, these samples showed no unusual results compared to samples consistently assigned to one species.

Sequencing was conducted on an Illumina HiSeq4000 platform (KAPA library preparation) and returned a mean coverage of about 25X 150bp paired end reads per individual. The quality of raw whole genome sequence data of all 36 samples was checked using FastQC v0.11.5 (Andrews 2010) in combination with MultiQC v1.5 (Ewels et al. 2016) to make comparison between samples easier and to decide for applicable trimmer.

Trimming was done using Trimmomatic v0.36 (Bolger et al. 2014) with the wrapper Autotrim v0.6 (Waldvogel et al. 2018) using the following trimmer and options:

ILLUMINACLIP:adapterfile:2:30:10:7:true CROP:148 SLIDINGWINDOW:4:20 MINLEN:50 TOPHRED33.

After trimming, quality was again checked using FastQC v0.11.5 (Andrews 2010) in combination with MultiQC v1.5 (Ewels et al. 2016). Duplication rate ranged from 7.3 to 20.7%, GC content was always about 32% and mean quality phred score was consistently above 30. Ultimately, we concluded that all sequencing resulted in good quality output and proceeded with mapping of all samples.

Mapping to the latest *C. riparius* reference genome (Schmidt et al. 2020) was conducted using BWA mem v0.7.15 (Li and Durbin 2009) applying the –M option for Picard (Broad Institute 2019) compatibility (for downstream duplication marking) and using default setting otherwise. Resulting SAM files were converted to BAM, sorted and indexed using SAMtools (Li et al. 2009). Resulting files were checked using Qualimap v2.2.1 (Okonechnikov et al. 2015). Between 87.6% and 97.3% (mean: 94.8%) of reads per sample were mapped in pair. Mean coverage across samples was 22.8 (min: 18.1, max: 30.4) with a mean mapping quality of 44.5 (min: 42.9, max: 45.41). Median insert size ranged from 271 – 308 bp (mean: 290). The (estimated) duplicated reads (mean: 28.6%) where flagged for downstream analysis using Picard MarkDuplicatesWithMateCigar (Broad Institute 2019) with default settings and VALIDATION_STRINGENCY=SILENT MINIMUM_DISTANCE=300. The resulting files were again indexed using Samtools (Li et al. 2009).

GATK v3.8.0 (Van der Auwera et al. 2013) was used for further data processing. To account for SNPs resulting from insertions and deletions (InDels) and therefore not being actual SNPs, we first applied local realignment to minimize the number of mismatches across all reads. For realignment we first created a target list using RealignerTargetCreator with default settings and then ran IndelRealigner with default settings as well.

The base quality scores produced by the sequencer are subject to numerous sources of non-random technical errors resulting in over- or under-estimation of said scores. We applied machine learning to account for this. For base quality recalibration we first identified shared SNPs per taxa by initially calling SNPs on all samples using GATKs UnifiedGenotyper to call high quality SNPs with a minimum phred score quality of 40 (-stand_call_conf 40) with respect to the latest *C. riparius* reference genome (Schmidt et al. 2020). Variants below the phred score threshold were filtered out. We used bcftools v1.9 isec (Li 2011) with default settings to identify shared SNPs among all *C. riparius* and *C. piger* samples, respectively. For grouping per taxon, we relied on the L44 and microsatellite data.

Base quality score recalibration per taxon was done using GATK (Van der Auwera et al. 2013) BaseRecalibrator with the identified shared SNPs used for the –knownSites option to create a taxa specific recalibration table. The resulting recalibration table was then used for the –BQSR option in the second round of using BaseRecalibrator. The recalibration was applied to the realigned BAM files using PrintReads and providing the recalibration table to the –BQSR option.

Good quality (-stand_call_conf 40) SNPs were called using GATKs (Van der Auwera et al. 2013) UnifiedGenotyper and all variants below that threshold filtered out. We additionally filtered for biallelic SNPs that were supported by at least 6 reads using vcftools with the –min-alleles 2, –max-alleles 2 and –min-meanDP 6 options. Afterwards, we used bcftools v1.9 (Li 2011) to only keep variants from regions with a coverage between 6-100 and which are supported by a mapping quality >30 (bcftools view -i 'MIN(INFO/DP>5) & MIN(INFO/DP<101) & MIN(INFO/MQ>30) & (INFO/AN=2)).

Ultimately, we merged all resulting files and additionally created taxa specific VCF files using bcftools v1.9 (Li 2011) merge with default settings. Due to merging creating new non-biallelic sites in the resulting file, we again filtered for biallelic sites using the same approach as above.

For ancestry inference it is recommended for both, PCA and ADMIXTURE (Alexander et al. 2009), to prune the dataset for highly linked SNPs to avoid overemphasising of their contribution to the taxa split. We did that by using the bcftools (Li 2011) plugin “prune” to prune all SNPs with a squared correlation coefficient of more than 0.8 (-l 0.8). Afterwards, we excluded all positions that were not genotyped for all 36 samples and calculated the PCA as well as ran ADMIXTURE (Alexander et al. 2009).

# 1.2. Identification of mutually isolated genomic regions – estimating the lower temporal detection limit

Even in the absence of gene-flow between the taxa due to strong purifying selection in certain parts of the genome, mutations must occur and increase in frequency after divergence in order to identify these parts as mutually isolated. This divergent mutation accumulation requires a certain amount of time, which depends on the mutation rate and the demography of the taxa in question. Therefore, the detectability of isolated windows by divergent SNPs has an inherent lower limit for the respective minimal divergence time.

We explored this lower temporal detection limit with a simulation, using the coalescence approach in SIMCOAL (Excoffier 2000). We used the mutation rate (µ = 2.1 x 10-9) and the effective population size (1.5 x 10^6^) estimated for *C. riparius* (Oppold and Pfenninger 2017) and assumed that these values apply for *C. piger* as well. We simulated 10kb windows with the above empirical parameter values. We simulated two sorts of windows:

i) Genomic windows shielded from gene-flow between the taxa by purifying selection (henceforth isolated windows). The isolated windows were simulated as completely isolated. Based on Foucault et al. (2018), we assumed that purifying selection after hybridisation occurs swiftly enough to justify this approximation. After exploratory simulations, we concentrated on divergence events that occurred between 0.7 and 1.5 N_e_ generations ago in 0.1 generation time steps (Suppl. Fig. 4a).

ii) Genomic windows with post-divergence gene-flow corresponding to the empirical gene-flow rate (called non-isolated windows). The non-isolated windows were simulated in 0.1 generation time steps as well, but until 9 N_e_ generations ago (Suppl. Fig. 4b). This time corresponds to the maximum divergence time empirically estimated for isolated windows, (see Results). The gene-flow rate among taxa for the simulation was estimated from the mean F_ST_ for non-isolated windows.

For each time step in both isolated and non-isolated windows, we simulated 5000 replicates, assuming constant population sizes through time. We sampled 7 and 29 individuals of *C. piger* and *C. riparius*, respectively, to mirror the empirical sample distribution. We recorded the proportion of divergent vs. shared SNPs from the two diverged populations and inferred the isolated – non-isolated status for each replicate as described above. We used these results to estimate the false negative rate (i.e. windows simulated as isolated that were not identified as such) and the false positive rate (i.e. non-isolated windows that were falsely flagged as isolated). By fitting a linear function, we estimated the divergence time by which the false positive rate reached zero. Likewise, we fitted a sigmoid function to model the probability of a window with given divergence time to be correctly flagged as isolated.

These simulations indicated that the false positive rate for identification of an isolated window is below 1 %, given the empirical gene-flow rate for the non-isolated parts of the genome. The false negative rate dropped to zero for windows simulated to have diverged 1.7 N_e_ generations ago and older (Suppl. Fig. 7a). Focussing on the estimated divergence time showed that the probability to correctly call a window as isolated was 50 % for divergence time estimates as recent as 0.1 N_e_ generations ago and reached >99 % for estimates of 0.2 N_e_ generations or more (Suppl. Fig. 7b).

# 1.3 Known reduction of recombination rate of chromosome III

In addition to comparing LD between isolated and non-isolated windows, we capitalised on the known reduction of recombination rate of chromosome III, which contains the sex-determining region (Schmidt et al. 2020). Using a χ²-test we determined whether windows from scaffolds known to be located on this chromosome are more often isolated than expected from the rest of the genome.

61 % of windows found on scaffolds known to be located on chromosome III with its reduced recombination rate were isolated. This is significantly more than expected from the rest of the genome (χ² = 15.545, d.f. = 1, p < 0.0001). This indicates that very low recombination rates indeed increase the chance of genomic isolation. Assumably, after having fallen below a certain threshold at which contradicting forces like selection are no longer equalised. However, the influence of the recombination rate in *Chironomus* in general remains low as shown by the applied linear model (see “Temporal divergence dynamics” in the manuscript).

# 1.4 Gene Ontology (GO) term enrichment analyses

Genes were assigned to be located on either isolated or non-isolated based on the PCA inference and their respective physical location. Genes falling across the border between isolated and non-isolated regions were assigned to the isolated region. GO-annotation of genes was performed using InterProScan v5.31-70.065, resulting in the annotation of 7,951 genes with a least one GO term. Enrichment was estimated using the weight01 algorithm and calculating Fisher statistics in topGO (Alexa and Rahnenführer 2018). As recommended in the topGO manual (Alexa and Rahnenführer 2018) we pruned the GO hierarchy from terms with less than 5 annotated genes to yield more stable results.

# 1.5. Historic divergence dynamics

To test our three predictions ((i) non-random size distribution of isolated regions, ii) isolated regions increase both in number and size over time and divergence time estimates should extend to the present, iii) size of isolated regions is roughly proportional to their divergence time, we used the following methods:

- To test the non-random size distribution, we randomly selected 4917 windows (corresponding to the number of empirically inferred isolated windows) from the entire genome. We joined adjacent windows to isolated regions and calculated the resulting mean length across all regions. This process was repeated 10,000 times to obtain a null distribution. We then determined the quantile rank of the observed mean isolated region length and compared it to the empirically found mean length.
- To infer the temporal isolation dynamics, we applied a molecular clock approach: Genetic distance between isolated windows among taxa was calculated using the command line version of MEGA X v10.0.5 (Kumar et al. 2018). This divergence estimate was then translated into generations in the past by applying the mutation rate estimate available for this taxon (Oppold and Pfenninger 2017) and scaled in multiples of Ne generations. We divided the period limited by the window with the highest estimated divergence time (~13 million generations = 9 Ne) into 27 bins of equal length of 1/3 Ne generations. We counted the number of observed windows with estimated divergence times falling in each of those bins. We then compared the resulting empirical frequency distribution with distributions resulting from the simulation of different divergence scenarios. However, due to the large variance associated with the coalescence and mutation process, it is not possible to directly compare the empirical distribution with these expected distributions. We therefore simulated 100,000 10 kb windows for each of 27 divergence times (0.33 to 9 Ne generations in 1/3 Ne steps) as described above. From each of these 27 simulation samples, we then randomly drew 4917 simulated divergence time estimates 100,000 times by randomly drawing n simulated divergence time estimates, with n corresponding to the number of expected divergence events for the respective bin of the respective divergence scenario (Fig. 1a). We took the detection limit for isolated windows (see above) into account by incorporating simulated divergence time estimates according to their probability of being correctly called. From the resulting set, equal in size to the observed number of isolation windows, we calculated a frequency distribution with the same bins as for the empirical divergence time distribution. We calculated skewness, kurtosis, standard deviation and mode of the resulting distributions. The mode was chosen to find the peak of the divergence time distributions of isolated regions indicating the point in time at which either full GI or stable steady-state equilibrium was reached. Skewness holds information about the asymmetry of the distribution and hints at the course of divergence through time. Kurtosis was calculated to obtain information about the speed at which divergence accumulated. The standard deviation shows how divergence times are spread out from the average values and whether divergence built up rather instantly or rather continuously. Mean values per summary statistic and model can be found in Suppl. Tab. 3. These summary statistics were subsequently compared to those of the empirical data using an Approximate Bayesian Computation (ABC) inference framework to eventually estimate the posterior probability of each model to yield the empirical data. ABC approaches circumvent the problem of calculating the computationally very costly likelihood function needed for classical statistical inference by using summary statistics and simulations to simplify the information contained in the data while simultaneously retaining maximum informativeness (Csilléry et al. 2010). Using the rejection method observed and simulated summary statistics were compared and models ranked by the Euclidian distance to the empirical data (Beaumont 2010). Based on this, the Bayes factor was calculated, representing the likelihood ratio of the marginal likelihood that the respective model is closest to the empirical data (Lee and Wagenmakers 2014). Analyses were carried out using the R package abc (Csilléry et al. 2012). In order to confirm the informativeness of the summary statistics chosen to distinguish the different models, we visually inspected them using boxplots. Additionally, we performed cross-validation for model selection to evaluate the ABC inference frameworks ability to distinguish between the five models by applying the *cv4postpr* function (tolerance rate = 0.01, method = rejection) to calculate a confusion matrix based on 100 samples (Suppl. Tab. 4). We proceeded by calculating the posterior probabilities of each model using the *postpr* function (tolerance rate = 0.01, method = rejection). The resulting Bayes factors for each model were interpreted according to Lee and Wagenmakers (2014) to identify the model with the most relative evidence to represent the true model.
- To test the hypothesis of divergence islands growing with time, we initially tested whether divergence time (represented by sequence divergence D_xy_ between *C. riparius* and *C. piger*), strength of selection (represented by the Watterson estimator θ) and physical linkage (r^2^) between pairs of SNPs differ between isolated and non-isolated regions. θ is a measure of genetic variation within a population and often described as the population mutation rate. It can be estimated using the Watterson estimator by dividing the number of segregating sites by the harmonic number of sequences minus 1. This means, that high estimated values indicate greater genetic variation compared to regions with lower estimated values. In regions under strong selection certain genotypes are favoured and/or deleterious alleles removed, both resulting in low genetic variation and therefore low estimates for θ. Therefore, θ may be used as a proxy for strength of selection. We proceeded by joining adjacent isolated windows to isolated regions, calculated their length and correlated it to its mean estimated divergence time, mean strength of selection and mean physical linkage. Normal distribution of data was obtained by log-transforming the length and boxcox transforming r^2^. We applied a linear regression model (LM) using length of isolated regions as dependent and D_xy_, θ and r^2^ as independent factors in R (R Core Team 2017). To determine each factors effect size we calculated η^2^ from the sum of squares in the applied type-II ANOVA (car package(Weisberg 2019)).

Owing to the small number of samples per population, we were unable to reliably calculate θ (as a proxy for selection, see Suppl. Info. 1.5) and r^2^ estimates on the basis of our individual WGS data. θ is known to vary substantially between *C. riparius* populations (Oppold and Pfenninger 2017). r2 estimates are only valid within one population since they are strongly influenced by population structure producing LD between actually unlinked loci due to merging samples with different genetic backgrounds (Mangin et al. 2012). That fact would have limited our approach to infer LD on a maximum of 13 individuals (MG population). To calculate LD on individual resequencing data, haplotype phasing is required. As haplotype phasing describes a process of statistical estimation, the results are more robust with increasing sample size. In view of applicable phasing tools, e.g. SHAPEIT (Delaneau et al. 2012) or BEAGLE (Browning and Browning 2007), our sample numbers are to low to allow for robust phasing without additional data not available for *C. riparius* (e.g. genetic map stating recombination rates between SNPs and reference haplotypes).We therefore estimated both parameters based on Pool-Seq data from the same German sample site we sampled in this study. In view of LD estimation using LDx (Feder et al. 2012), this approach did not require haplotype phasing and at the same time increased the number of individuals considered to 100 making the analysis more robust. The limitation of the method to derive linkage only up to the read length proved to be negligible for our model since LD decayed much faster than within 250 bp (Suppl. Fig. 3).

# 1.6. Processes driving divergence

To compare the synonymous to non-synonymous variation in the isolated to the non-isolated regions we calculated McDonald-Kreitman Test (MKT) for non-isolated genes in the same way as for isolated genes (see “Processes driving divergence” in the manuscript). However, this may only be seen as proof of principle since the non-isolated regions violate a fundamental assumption of MKT: MKT compares the intraspecific variation with the interspecific variation (McDonald and Kreitman 1991). Interspecific variation can only occur after divergence and therefore requires selection to act simultaneously in different directions in two species (Rundle and Nosil 2005) without strong enough gene flow to compensate for this. In our study, non-isolated regions are defined by being not significantly different between the two *Chironomus* taxa and even appear to be freely exchangeable between them. Therefore, MKT is only applicable when arbitrarily assuming a divergence in the non-isolated genomic regions (Murga-Moreno et al. 2019).

For the 5916 genes in the non-isolated regions, MKT showed a significant accumulation of either synonymous or non-synonymous divergent SNPs for 53 of them. Neutrality index indicated that 35 of these were under negative selection, while 18 showed signs of positive selection. Compared to the isolated regions (451 significant genes, of which 421 are under negative selection and 30 under positive selection), these numbers are significantly low (Chi² = 279.366, p < 0.0001). Reasons for finding divergently selected genes in non-isolated parts, might be the window approach: The windows are arbitrarily limited and may partially contain only parts of genes or multiple genes with conflicting signals.

# Supplementary Figures


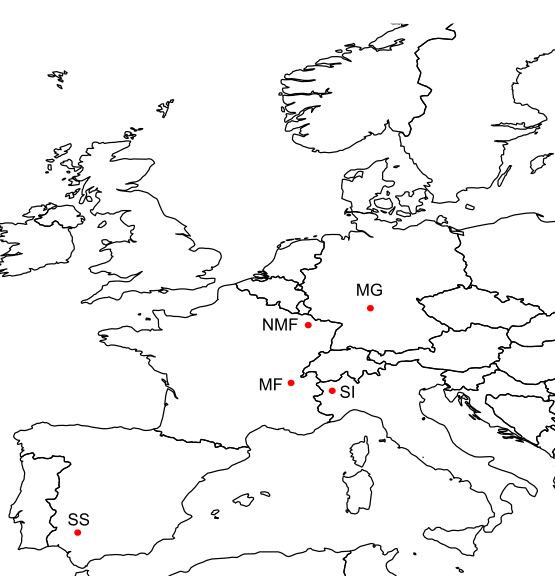


Supplementary Figure 1: Map of Europe displaying sample sites: MG: Hesse, Germany (50.188297, 9.214170); NMF: Lorraine, France (49.008298, 6.223934); MF: Lyon, France (46.017340, 4.911689); SI: Piemont, Italy (45.248064, 7.610818); SS: Andalusia, Spain (37.807905, -4.765055).


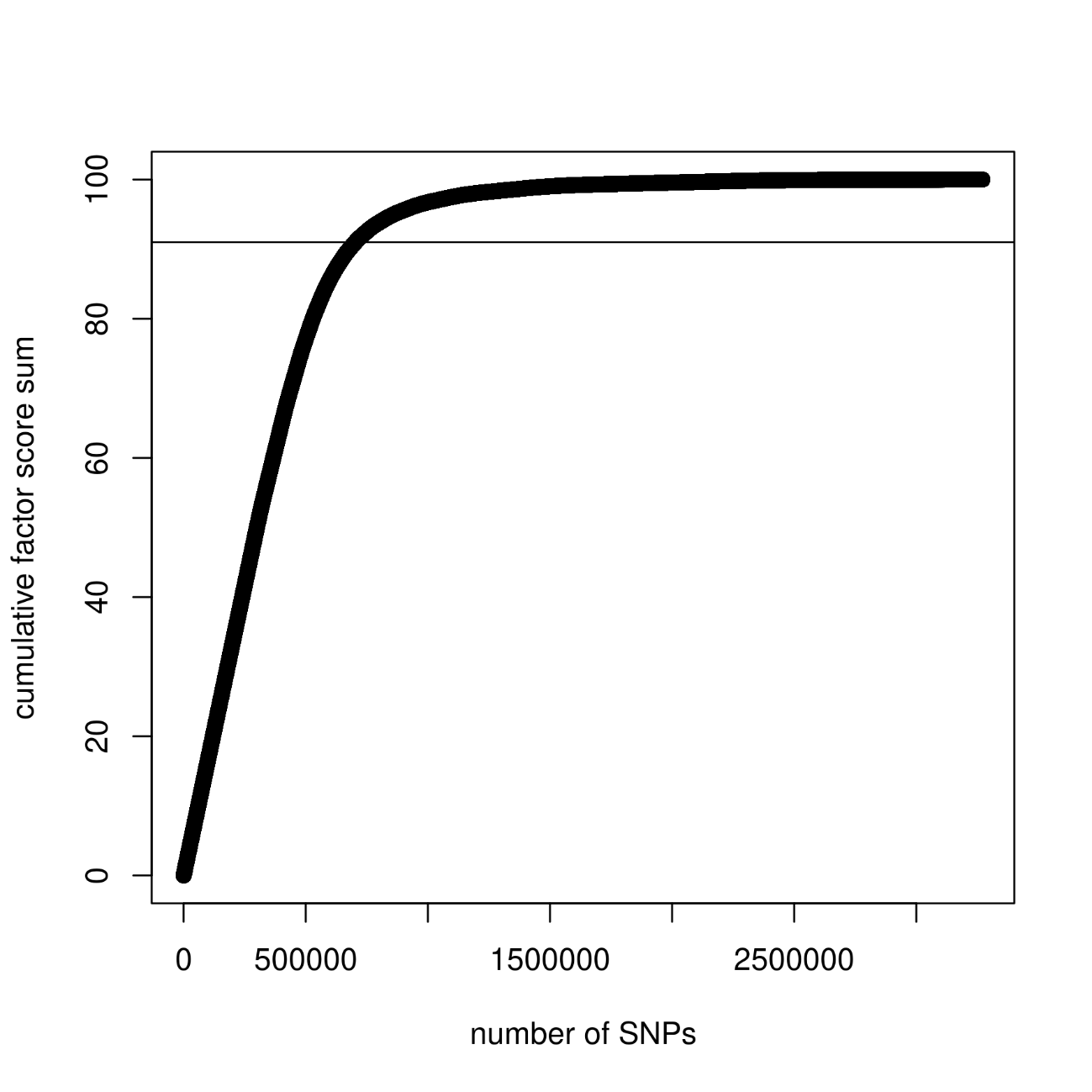


Supplementary Figure 2: Cumulative factor score sum of SNPs associated with (species splitting) PC1 in the PCA used to identify the isolated windows (see Fig. 2a). All SNPs below the horizontal line (y=91, (n = 700,000 SNPs), showing linear increase in cumulative factor score sum, were flagged as sepSNPs while the ones above it were flagged as resSNPs, since they made only minimal contributions to the splitting of the species.

Supplementary Figure 3: LD decay curve of maximum likelihood estimates of r2. Points represent the average r2 within distance classes. Averages were applied only to pairs that had minor allele frequency >0.2. The decay model was fit in SciPy 1.5.2.

Supplementary Figure 4: Experimental design for coalescence simulations. a) Simulation of non-isolated 10 kb windows. A divergence time corresponding to the empirical mean divergence time (3.06 Ne generations) was assumed and a per generation gene-flow of Nm = 1/(4F_ST_ + 1) with the empirical mean F_ST_ among non-isolated windows of 0.24. b) Simulation of isolated 10 kb windows with divergence times corresponding to 0.33 to 9 Ne generations with 0.33 generation steps. The sample size corresponded to the real sample size (seven for C. piger and 29 for C. riparius).

Supplementary Figure 5: Maximum likelihood estimation of individual ancestry based on 900345 SNPs (quality filtered, LD-pruned) using ADMIXTURE v1.3.0 with K=2.


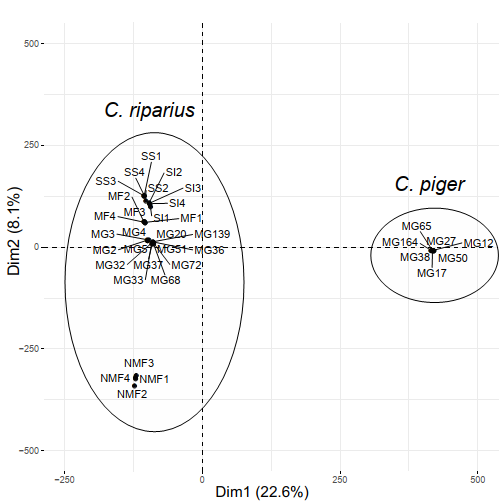


Supplementary Figure 6: Principal component analysis of the LD-pruned SNP data set. Axis one, representing 22.6% of total variation, separated the two taxa as identified from microsatellite and mitochondrial marker loci.

Supplementary Figure 7: Inference of lower temporal resolution limit with coalescence simulations. a) False negative detection rate for 10 kb windows simulated as isolated in relation to the simulated divergence time. The regression line reaches zero at 1.701 N_e_ generations. b) Probability for a 10 kb window simulated as isolated with an estimated divergence time estimate to be correctly flagged as such. The fitted function follows the equation y = 1 / 1 + 883.17*e^(-4.1205E-05x)^.

Supplementary Figure 8: Influence of fluctuating population size on distribution of Tajima’s D. One hundred 10 kb windows were simulated with Simcoal as described in the main manuscript for i) a constant population size of 1.5 mio individuals and ii) a population of initially equal size which grew exponentially (N_t_ = N_0_ * e^(rt)^) for 10 generations with an r of 0.001, was then reduced in a single generation to the initial size. This boom-bust cycle was repeated 5,000 times (i.e. for 50,000 generations). Resulting Tajima’s D was calculated with Arlequin 3.5. The difference between both distributions was highly significant (p < 0.001).


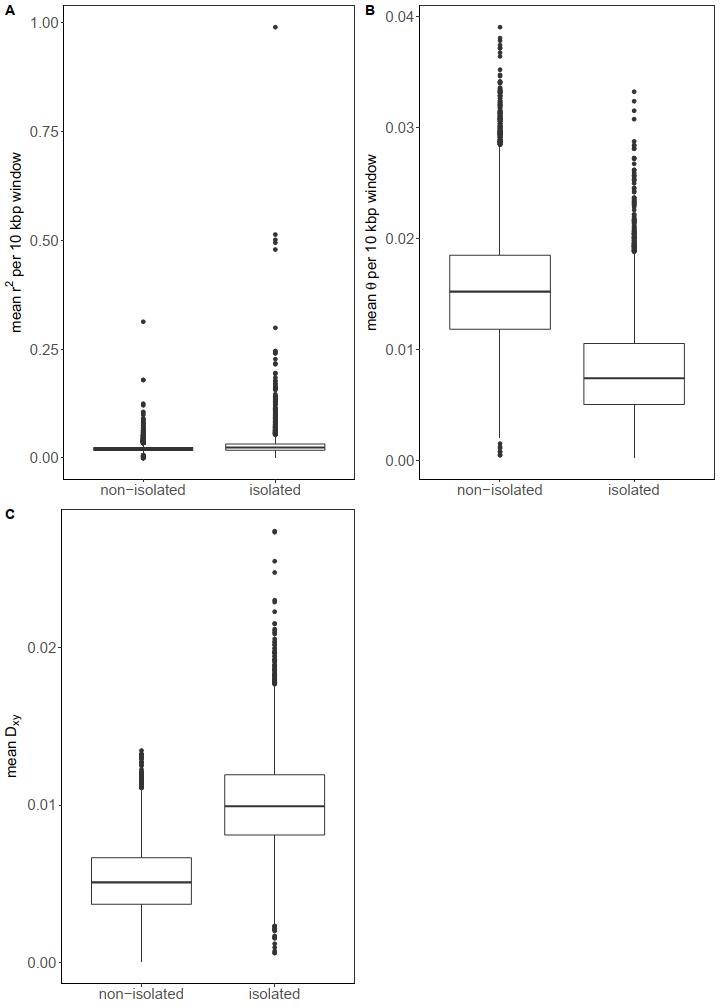


Supplementary Figure 9: Comparisons of non-isolated vs. isolated parts of the genome in view of factors influencing the length of isolated regions. a) Box-plots of short-scale linkage represented among non-isolated and isolated 10 kb windows measured as r^2^ for the German population (Pfenninger 2020). The difference of means (0.022 and 0.029, respectively) was highly significant (p < 0.001, Cohens’s d = 0.44). b) Box-plots of difference in strength of selection measured as θ among non-isolated and isolated windows in the German population (Pfenninger 2020). The difference of means (0.015 and 0.008, respectively) was highly significant (p < 0.001, Cohen’s d = -1.44). c) Box-plots of Dxy for non-isolated and isolated windows between the two taxa. The differences in of means (0.005 and 0.01, respectively) was highly significant (p < 0.001, Cohen’s d = 2).

# Supplementary Tables

Supplementary Table 1: Barcoding results for all 36 samples based on universal mitochondrial COI (Folmer et al. 1994), taxa specific nuclear L44 (Oppold et al. 2016) as well as microsatellites markers. Sample names refer to the respective sample site: MF = Lyon, France (45.76404, 4.83565), MG = Hasselbach, Germany (50.18816, 9.21423), NMF = Lorraine, France (48.87442, 6.20809), SI = Piemont, Italy (45.05223, 7.51538) and SS = Andalusia, Spain (37.54427, -4.72775). In addition, number of non-LD pruned but quality filtered SNPs per sample are given.

*Samples obtained from Waldvogel et al. (Waldvogel et al. 2018)

†Samples newly sequenced for this study originating form a German population with known hybridization history (Foucault et al. 2018), sampled between June and September 2016

| Sample | COI | L44 | microsatellite assignment | SNPs |
| --- | --- | --- | --- | --- |
| MF1^*^ | riparius | riparius |  | 667797 |
| MF2^*^ | riparius | riparius |  | 678676 |
| MF3^*^ | riparius | riparius |  | 661929 |
| MF4^*^ | riparius | riparius |  | 677490 |
| MG139^†^ | riparius | riparius | riparius | 751886 |
| MG2^*^ | riparius | riparius | riparius | 678341 |
| MG20^†^ | riparius | riparius | riparius | 752714 |
| MG3^*^ | riparius | riparius | riparius | 663767 |
| MG36^†^ | riparius | riparius | riparius | 740996 |
| MG37^†^ | riparius | riparius | riparius | 747781 |
| MG4^*^ | riparius | riparius | riparius | 662877 |
| MG5^*^ | riparius | riparius | riparius | 666850 |
| MG68^†^ | riparius | riparius | riparius | 747256 |
| MG32^†^ | piger | riparius | riparius | 733482 |
| MG33^†^ | piger | riparius | riparius | 753495 |
| MG51^†^ | piger | riparius | riparius | 739065 |
| MG72^†^ | piger | riparius | riparius | 755869 |
| NMF1^*^ | riparius | riparius |  | 593115 |
| NMF2^*^ | riparius | riparius |  | 612021 |
| NMF3^*^ | riparius | riparius |  | 646323 |
| NMF4^*^ | riparius | riparius |  | 645149 |
| SI1^*^ | riparius | riparius |  | 712444 |
| SI2^*^ | riparius | riparius |  | 697992 |
| SI3^*^ | riparius | riparius |  | 685526 |
| SI4^*^ | riparius | riparius |  | 695357 |
| SS1^*^ | riparius | riparius |  | 678860 |
| SS2^*^ | riparius | riparius |  | 719059 |
| SS3^*^ | riparius | riparius |  | 704670 |
| SS4^*^ | riparius | riparius |  | 666729 |
| MG164^†^ | piger | piger | piger | 1610621 |
| MG27^†^ | piger | piger | piger | 1636057 |
| MG38^†^ | piger | piger | piger | 1640623 |
| MG50^†^ | piger | piger | piger | 1608025 |
| MG12^†^ | riparius | piger | piger | 1569707 |
| MG17^†^ | riparius | piger | piger | 1586240 |
| MG65^†^ | riparius | piger | piger | 1592807 |

Supplementary Table 2: Gene ontology (GO) term enrichment analysis of positively selected genes found in isolated parts of the genome. Significantly (p < 0.05) overrepresented GO-Terms in the categories biological process (BP) and cellular component (CC) for isolated regions are shown. The column Annotated shows the number of genes annotated with the respective GO term, the column Observed the number of these genes in the isolated region and the column Expected the number expected if these genes were equally distributed over the entire genome. Probability values for the number of observed genes being within random expectations are based on the weight Fisher algorithm

| Category | GO ID | Term | Annotated | Observed | Expected | p-value |
| --- | --- | --- | --- | --- | --- | --- |
|  |  |  |  |  |  |  |
| BP | 0046854 | phosphatidylinositol phosphorylation | 8 | 1 | 0.02 | 0.02 |
| BP | 0008299 | isoprenoid biosynthetic process | 9 | 1 | 0.02 | 0.022 |
| BP | 0006413 | translational initiation | 13 | 1 | 0.03 | 0.032 |
| CC | 0016592 | mediator complex | 23 | 1 | 0.05 | 0.045 |

Supplementary Table 3: Mean values of all 4 summary statistics per divergence scenario describing the divergence time distributions used in the ABC model inference approach as well as the empirical data.

|  | Continuously | Episodic | Eruption | Escalating | Instant | empirical |
| --- | --- | --- | --- | --- | --- | --- |
| Mode | 9,308,528 | 3,522,505 | 9,500,000 | 0 | 3,727,452 | 4,000,000 |
| Skewness | 33.75377 | 1.103289 | -0.472158 | 57.81606 | 1.25022 | 0.2676025 |
| Kurtosis | 2,122.895 | 3.448958 | 87.45977 | 3,636.169 | 3.363856 | 0.7621875 |
| Standard deviation | 2.155438e+14 | 1,192,308 | 9,547,392,745 | 3.970017e+12 | 1,099,067 | 1,588,178 |

| Supplementary Table 4: Confusion matrix based on leave-on-out cross validation for 100 samples for each of the 5 models, applying a tolerance rate of 0.01 and using the “rejection” method. | Continuously | Episodic | Eruption | Escalating | Instant |
| --- | --- | --- | --- | --- | --- |
| Continuous | 87 | 0 | 0 | 13 | 0 |
| Episodic | 0 | 100 | 0 | 0 | 0 |
| Eruption | 5 | 0 | 95 | 0 | 0 |
| Escalating | 11 | 0 | 0 | 89 | 0 |
| Instant | 0 | 3 | 0 | 0 | 97 |

# Supplementary Code

Below an exemplary SIMCOAL input file is shown. The other files differ solely by the respective divergence time.

*//Parameters for the coalescence simulation program: simcoal.exe*

*2 samples to simulate*

*//Population effective sizes (number of genes)*

*1500000*

*1500000*

*//Samples sizes*

*58*

*14*

*//Growth rates: negative growth implies population expansion*

*0*

*0*

*//Number of migration matrices: 0 implies no migration between demes*

*0*

*//historical event: time, source, sink, migrants, new deme size, new growth rate, migration matrix index*

*1 historical event*

*1050000 0 1 1 1 0 0*

*//Mutation rate per generation for the whole sequence*

*0.000021*

*//Number of nucleotides to simulate*

*100000*

*//data type either DNA, RFLP, or MICROSAT: If DNA, we need a second term for the transition bias*

*DNA 2*

*//Gamma parameter (if 0: even mutation rates, if >0: shape parameter of the Gamma distribution*

*0 // Second parameter is the number of discrete rate categories to simulate: if zero: continuous distribution*

Alexa, A., and J. Rahnenführer. 2018. topGO: Enrichment Analysis for Gene Ontology.

Alexander, D. H., J. Novembre, and K. Lange. 2009. Fast model-based estimation of ancestry in unrelated individuals. Genome Res. 19:1655–1664.

Andrews, S. 2010. FAstQC A Quality Control Tool for High Throughput Sequence Data.

Beaumont, M. A. 2010. Approximate Bayesian computation in evolution and ecology. Annu. Rev. Ecol. Evol. Syst. 41:379–406.

Bolger, A. M., M. Lohse, and B. Usadel. 2014. Trimmomatic: A flexible trimmer for Illumina sequence data. Bioinformatics 30:2114–2120.

Broad Institute. 2019. Picard toolkit.

Browning, S. R., and B. L. Browning. 2007. Rapid and accurate haplotype phasing and missing-data inference for whole-genome association studies by use of localized haplotype clustering. Am. J. Hum. Genet. 81:1084–1097.

Csilléry, K., M. G. B. Blum, O. E. Gaggiotti, and O. François. 2010. Approximate Bayesian Computation (ABC) in practice. Trends Ecol. Evol. 25:410–418.

Csilléry, K., O. François, and M. G. B. Blum. 2012. Abc: An R package for approximate Bayesian computation (ABC). Methods Ecol. Evol. 3:475–479.

Delaneau, O., J. Marchini, and J.-F. Zagury. 2012. A linear complexity phasing method for thousands of genomes. Nat. Methods 9:179–81.

Ewels, P., M. Magnusson, S. Lundin, and M. Käller. 2016. MultiQC: Summarize analysis results for multiple tools and samples in a single report. Bioinformatics 32:3047–3048.

Excoffier, L. 2000. Computer note. SIMCOAL: a general coalescent program for the simulation of molecular data in interconnected populations with arbitrary demography. J. Hered. 91:506–509.

Feder, A. F., D. A. Petrov, and A. O. Bergland. 2012. LDx: Estimation of Linkage Disequilibrium from High-Throughput Pooled Resequencing Data. PLoS One 7:1–7.

Folmer, O., M. Black, W. Hoeh, R. Lutz, and R. Vrijenhoek. 1994. DNA primers for amplification of mitochondrial cytochrome c oxidase subunit I from diverse metazoan invertebrates. Mol. Mar. Biol. Biotechnol. 3:294–299.

Foucault, Q., A. Wieser, C. Heumann-Kiesler, J. Diogo, B. Cocchiararo, C. Nowak, A. Waldvogel, and M. Pfenninger. 2018. An experimental assessment of reproductive isolation and its consequences for seasonal hybridization dynamics. Biol. J. Linn. Soc. 1–11.

Kumar, S., G. Stecher, M. Li, C. Knyaz, and K. Tamura. 2018. MEGA X: Molecular evolutionary genetics analysis across computing platforms. Mol. Biol. Evol. 35:1547–1549.

Lee, M. D., and E.-J. Wagenmakers. 2014. Baysian Cognitive Modeling - A Pracitcal Course. Cambridge University Press, Cambridge.

Li, H. 2011. A statistical framework for SNP calling, mutation discovery, association mapping and population genetical parameter estimation from sequencing data. Bioinformatics 27:2987–2993.

Li, H., and R. Durbin. 2009. Fast and accurate short read alignment with Burrows-Wheeler transform. Bioinformatics 25:1754–1760.

Li, H., B. Handsaker, A. Wysoker, T. Fennell, J. Ruan, N. Homer, G. Marth, G. Abecasis, and R. Durbin. 2009. The Sequence Alignment/Map format and SAMtools. Bioinformatics 25:2078–2079.

McDonald, J. H., and M. Kreitman. 1991. Adaptive protein evolution at the Adh locus in Drosophila. Nature 351:652–654.

Murga-Moreno, J., M. Coronado-Zamora, S. Hervas, S. Casillas, and A. Barbadilla. 2019. IMKT: The integrative McDonald and Kreitman test. Nucleic Acids Res. 47:W283–W288.

Okonechnikov, K., A. Conesa, and F. García-Alcalde. 2015. Qualimap 2: Advanced multi-sample quality control for high-throughput sequencing data. Bioinformatics 32:292–294.

Oppold, A.-M., J. A. M. Pedrosa, M. Bálint, J. B. Diogo, J. Ilkova, J. L. T. Pestana, and M. Pfenninger. 2016. Support for the evolutionary speed hypothesis from intraspecific population genetic data in the non-biting midge chironomus riparius. Proc. R. Soc. B Biol. Sci. 283.

Oppold, A.-M., and M. Pfenninger. 2017. Direct estimation of the spontaneous mutation rate by short-term mutation accumulation lines in Chironomus riparius. Evol. Lett. in press.

R Core Team. 2017. R: A language and environment for statistical computing. R Foundation for Statistical Computing, Vienna, Austria.

Rundle, H. D., and P. Nosil. 2005. Ecological speciation. Ecol. Lett. 8:336–352.

Schmidt, H., A. Waldvogel, S. L. Hellmann, T. Hankeln, and M. Pfenninger. 2020. A high-quality genome assembly from short and long reads for the non-biting midge Chironomus riparius ( Diptera ). G3 Genes, Genomes, Genet. 10.

Van der Auwera, G. A., M. O. Carneiro, C. Hartl, R. Poplin, G. del Angel, A. Levy-Moonshine, T. Jordan, K. Shakir, D. Roazen, J. Thibault, E. Banks, K. V. Garimella, D. Altshuler, S. Gabriel, and M. A. DePristo. 2013. From fastQ data to high-confidence variant calls: The genome analysis toolkit best practices pipeline.

Waldvogel, A. M., A. Wieser, T. Schell, S. Patel, H. Schmidt, T. Hankeln, B. Feldmeyer, and M. Pfenninger. 2018. The genomic footprint of climate adaptation in Chironomus riparius. Mol. Ecol. 27:1439–1456.

Weisberg, F. J. 2019. An R Companion to Applied Regression. SAGE, Thousand Oaks CA.
